# Towards High Throughput Structuring of Liquid Foams in Microchannels: Effect of Geometry, Flowrate and Formulation

**DOI:** 10.3390/mi12111415

**Published:** 2021-11-18

**Authors:** Julian Sepulveda, Agnès Montillet, Dominique Della Valle, Catherine Loisel, Alain Riaublanc

**Affiliations:** 1 Oniris, CNRS, GEPEA, Université de Nantes, UMR 6144, F-44322 Nantes, France; julian.sepulveda@univ-nantes.fr; 2 ONIRIS, F-44000 Nantes, France; dominique.dellavalle@oniris.fr (D.D.V.); catherine.loisel@oniris.fr (C.L.); 3 UR 1268 Biopolymères, Interactions, Assemblages, INRAE, T-44000 Nantes, France; alain.riaublanc@inrae.fr

**Keywords:** food foams, multiphase flow, non-Newtonian fluid, bubble breakup, microfluidics, high speed imaging, flow typology

## Abstract

This work is part of a study aiming to design a high-throughput foaming microsystem. The main focused field of application is the food industry. With the objective of improving the design of the microdevice, the effects of the geometry and the nature of the liquid base are presently investigated through visualizations of the flow typology of bubbles trains, aiming to expand the knowledge on key parameters that lead to an improved gas breakup. The tested set of conditions is not encountered in traditional microfluidics systems: i.e., throughputs up to 19 L·h^−1^ for the liquid phase, process velocities around 20 m·s^−1^ and flow of complex fluids. The behavior of solutions based on xanthan gum (XG) and whey proteins (WPI) is compared to that of solutions containing one of these ingredients or other ones (caseinates, glycerol). The structural and end-used properties of the final foams, namely the bubble diameter and rheological behavior, are evaluated. The incorporation of XG induces bubble shape stabilization even at the highest shear rates (~10^5^ s^−1^) encountered in the mixing channel. “Controlled” interfacial breakup by tip-streaming or binary breakup are only observed with the WPI/XG biopolymers. This study indubitably highlights the essential role of the process/formulation interaction in the development of structural and functional properties of food foams when using microfluidics at high throughput.

## 1. Introduction

Foams are two-phase systems consisting of bubbles dispersed in a continuous liquid matrix. The properties deriving from this bi-phasic structuration make them highly useful in several industrial domains such as food industry, cosmetics and pharmaceutics [1,2]. The gas volume fraction, the size and size distribution of bubbles are the structural parameters governing the end properties of these systems [3,4,5]. For a food foam, the target void fraction is usually in the range of 0.5 to 0.9, and bubbles sizes are commonly between 0.1 and 3 mm [6]. The interaction between the formulation and process conditions determines the final foam structure [2,7,8].

Foam production can be performed using different methods, usually subdivided into continuous or discontinuous processes. Beating, a discontinuous process, is mostly encountered in artisanal foam production [9]. This method suffers from serious disadvantages, since it does not allow one to control the rate at which air is incorporated [7], and it has a rather high energy consumption.

Continuous technologies such as scraped surface exchangers and rotor-stators are mainly used in industrial settings [7]. The former are almost exclusively used for ice cream manufacturing, while a rotor-stator is a versatile device used for most industrially manufactured foams [4]. Despite its widespread use in industry, the rotor-stator technology exhibits some limitations due to the possible overheating of the product [10]. The use of microfluidic technologies appears as a promising alternative for continuous foam production. Indeed, microchannels have been found to offer interesting advantages similar to those yielded by intensified processes such as enhancing mixing operations and improving heat and mass transfer. Cheaper manufacturing and operating costs are also cited as an advantage [11,12,13]. Additionally, in colloidal dispersions, the co-injection of two fluids through microchannels has been reported to result in monodisperse bubbles [14] and droplets [15]. However, most works concerning microfluidics imply the use of very low flowrates, i.e., of the order of some milliliters per minute [16,17,18]. These flow conditions offer highly controlled conditions but are not relevant for industrial applications, in particular for the food industry. On the other hand, bi-phasic gas–liquid microfluidics studies implementing non-Newtonian fluids are rare [19].

It might be emphasized that continuous foaming processes usually work under moderate pressures but need a controlled depressurization of the gas–liquid mixture up to the atmospheric pressure. A too sudden variation of the pressure and/or of the velocity can induce the coalescence of bubbles and thus compromise the quality of the produced foams. Thus, the process lines generally include a relaxation system. This can be a long tube with eventual successive increases in diameter until the release of the foam at room pressure. These systems are generally empirically designed, but can be considered as an essential part of the whole process, as they help to secure the quality of the foam elaborated in the main part of the device.

This work is part of a general study that intends to design a miniaturized foaming device operating at high throughput, so as to meet some industrial conditions. A previous work gave a first insight on the genesis of bubble trains in a cross-slot microdevice at high throughput with non-Newtonian fluids formulated with biosourced molecules [20], and also focused on the potential of the obtained foams in view of food applications [21]. The whole process line was of a microdevice composed, the main function of which was to form the bubble train and also to increase the breakup, and secondly that of a relaxation line. It was noticed that the mean size of the bubbles in the foam collected at the outlet of the process line was smaller than the normalized size of bubbles visualized inside the mixing channel of the microdevice. The normalized size is that calculated at atmospheric pressure, from the size measured in the mixing microchannel and the corresponding pressure. This meant that other phenomena occurred, somewhere within the exit of the microdevice (outlet ports) and/or in the relaxation line. As it was not possible to visualize the flow inside the outlet ports of the microdevice, nor in the relaxation line, it was decided to mimic singularities that may be responsible for further breakup in these unobservable areas. A second study then specifically focused on the analysis of the evolution of the bubbles inside microdevices of various designs, in order to elucidate and characterize the mechanisms of bubble deformation and break-up at very high velocities (~2–20 m·s^−1^) and flow rates (10–20 L/h) [22]. In particular, the effect of geometric singularities such as a sudden enlargement of the outlet channel on the bubbles was observed and analyzed. In these former investigations, implemented aqueous model solutions were based only on the following biopolymers: xanthan gum (XG), as a thickener, and whey proteins (WPI), as a surfactant. It was shown that the use of high throughputs induces very high shear rates inside the channels, leading to a significant change in the viscosity of the tested solutions. The main insights of these studies concerned the observation and description of breakup mechanisms. It was shown that additional local elongational flow occurs in the geometrical expansion of the outlet microchannel, leading to more intensified bubble breakup.

One of the aims of the present study is thus to extend the poor existing knowledge on flow typologies of gas–liquid flows at high throughputs in microchannels, so as to help to select the optimal conditions for processing. In particular, this contribution intends to investigate the effect of the liquid phase’s formulation on the two-phase flow typology and bubble breakup inside the microchannel. With this objective, the experiments performed are based on varying the flow rate and the design of the device, in particular including an expansion area in the outlet microchannel. Some properties of solutions are also varied, such as the surface activity and the nature of the rheological properties in order to hierarchize their respective effects. In parallel, structural and functional properties of produced food foams, such as the mean diameter of bubbles and the foam viscosity and rigidity, are determined so as to link the impact of the process parameter and the formulation to the quality of final products (firmness and fineness).

## 2. Materials and Methods

### 2.1. Experimental Setup

**Figure 1:**
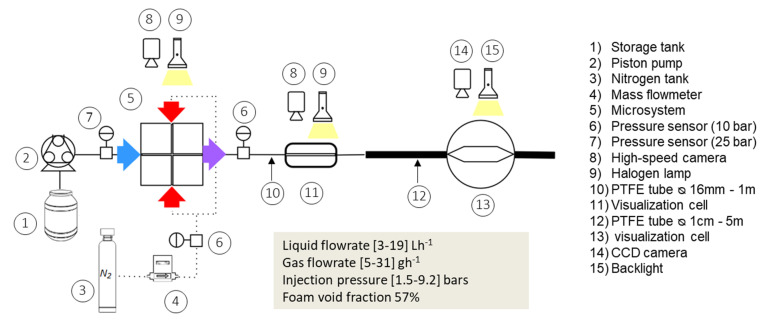
Schematic representation of the experimental processing line.

The experimental setup used for the study is represented schematically in Figure 1. The foaming solutions (1) were pumped using a reciprocating three-piston pump from Armen instruments (2). In parallel, gaseous nitrogen N_2_ (3) was regulated and transported using a mass flowmeter EL-FLOW Prestige Bronkhorst (4). The gas and the liquid base were mixed inside the microfluidic device (5). The injection pressure (*p_inj_*) and the outlet pressure in the microchannel were measured by 2 pressure sensors Gems 3100 series (6) with an operating range of 1 to 10 bar (all pressures are given in relative value from the ambient pressure). In the result section, Δ*p*_1_ corresponds to the pressure loss measured between these two pressure sensors. A third pressure sensor (7) with an operating range between 0 and 25 bar was placed in the solution supply line upstream from the microsystem in order to check the pressure values provided by the three-piston pump (Armen Instruments) (*p_pump_*). The two-phase gas–liquid flow formed inside the microchannels was then visualized using a high-speed camera HighSpeedStar 6 from LaVision (8) and a halogen lamp OSRAM 12 V 50 W (9) as a backlight source. The formed bubble train exited from the microfluidic device through a relaxation line composed of a polytetrafluoroethylene (PTFE) tube (10) with an internal diameter of 0.16 cm and a 1 m length. A first visualization cell (11) was designed to capture the state of the two-phase flow in this part of the processing line. For this purpose, this cell had a square section with an identical surface area section to that of the PTFE tube. However, since no significant flow phenomena were observed at this point, these results will not be provided in the present publication. Then, a gradual diffuser ensured the passage of the train of bubbles into a second PTFE tube (12) with an internal diameter of 1 cm and a length of 5 m. This allowed, as stated in the introduction, progressive flow relaxation as well as a reduction in pulsations from the pump. Finally, a second visualization cell (13) was placed at the end of the processing line in order to trap the foam for the evaluation of the bubble size distribution through image analysis. For this purpose, a CCD camera Sony XCL-5005 (14) with a resolution of 5 M pixels was used, along with a ring backlight source Schott KL 1500LCD (15).

### 2.2. Microfluidic Devices

**Figure 2:**
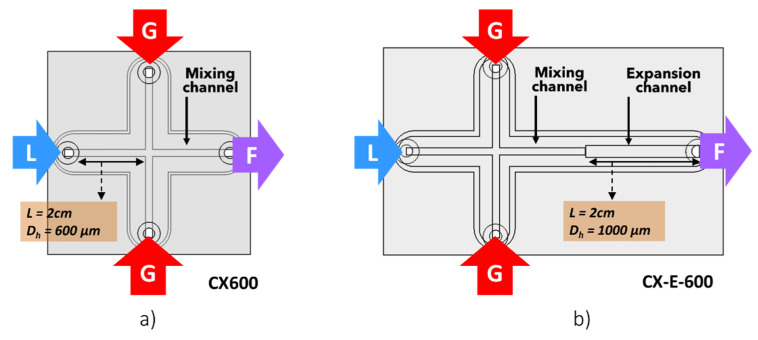
Schematic representation of the microfluidic devices. (**a**) reference device (CX600). (**b**) device with the same cross-shaped configuration, but with an integrated expansion channel downstream from the mixing channel (CX-E-600). Liquid solution (L), N_2_ (G), and gas–liquid dispersions which turn into foam along the processing line (F).

The two microfluidic devices (respectively introduced and designated as part 5 in Figure 1) used in this study are presented in Figure 2. Both had a cross-shaped arrangement of four squared microchannels with a 600 µm hydraulic diameter and lengths of 2 cm. Both devices were manufactured by milling one polymethyl methacrylate (PMMA) plate. A second PMMA plate was fixed to the first one using M10 screws and a seal to prevent any leakage.

The liquid base (L) was injected through one of the microchannels, whereas the gas (G) was injected from two inlets at a 90° angle to the liquid inlet. In such systems and geometries, the liquid acts as a plug in the main direction of flow. The gas was alternately split at each gas inlet by the liquid phase, releasing bubbles with a regular frequency [20]. The formed train of bubbles ran along the fourth microchannel (F) of the device before leaving it. This fourth channel is referred to as the ‘mixing channel’ in the following.

Figure 2a presents a schematization of the first device employed in this research (CX600). It has the typical cross-shaped geometry described above.

Concerning the second device (CX-E-600), shown in Figure 2b, it presented the same cross-shaped design, but it also incorporated a squared abrupt expansion with a hydraulic diameter of 1000 µm and a length of 2 cm which is set in the mixing microchannel. It is important to highlight that the abrupt expansion was not centered and that its upper side was aligned with that of the channel that preceded it. This abrupt enlargement of the microchannel was introduced so as to visualize the flow phenomena that may occur in the outlet port of the microsystems. Indeed, the outlet port introduced a sudden expansion to a section corresponding to 1 mm in diameter. As mentioned in the introduction, it is not possible to visualize the flow in the outlet port, and it has been shown that particular flow phenomena may develop within it. One of our previous works [22] demonstrated the key role played in breakup by the enlargement section in the CX-E-600 microsystem, when implemented with WPI-xanthan solutions. It was used here to investigate different fluids so as to determine whether the enlargement has the same effect or not on fluids with different properties.

### 2.3. Foaming Solutions

This work targeting food applications, the model liquid bases tested were formulated with biosourced molecules. Whey protein isolates (WPI) were used as surface active agents to stabilize the gas–liquid interfaces. The concentration of WPI was set at 3% (*w*/*w*) in order to ensure saturation at the interface. Xanthan gum (XG) was also added to the solutions. Its high viscosity at rest makes this hydrocolloid ideal for enhancing foam stabilization. Two concentrations for xanthan gum were used, 0.2% and 0.4% *w*/*w*. The solutions were named according to the WPI and xanthan concentration (WPI3XG02 and WPI3XG04). For the preparation procedure, the dry ingredients were mixed and then poured gently into a vessel containing distilled water while simultaneously being mixed with an overhead stirrer RZR 2020 from Heidolph instruments. The solutions were used immediately after preparation. Other solutions were prepared in order to evaluate the effect of the formulation on the two-phase flow inside the microfluidic devices, as well as to better understand the role of the selected biopolymers (WPI and XG) on the visualized phenomena. Additional solutions included: a solution containing only xanthan gum at 0.4% *w*/*w* (XG04), a solution using only WPI at 3% *w*/*w* (WPI3), one of the model solutions supplemented with 1.2% *w*/*w* of NaCl (WPI3XG04-NaCl), one of the model solutions in which WPI was replaced with another biosourced surfactant, namely sodium caseinates (CAS3XG04), pure distilled water, and finally an aqueous glycerol solution at 50% *w*/*w* to test a Newtonian solution with rather high viscosity.

### 2.4. Solution Characterization

#### 2.4.1. Density

The density of the solutions was measured using the Archimedes principle. This principle establishes that a fluid exerts on any immersed solid an upward vertical force equal to the weight of the fluid displaced. One and a half liters of the prepared solution was poured into a two-liter beaker and weighed (*m*_1_) using a balance PS 3500 R2 (Radwag) with a precision of ±0.01 g. Then, a suspended solid cylinder with a volume *V_obj_* = 136 ± 0.2 cm^3^ was immersed into the solution and the new mass was recorded as *m*_2_. Finally, the density of the solution ($\rho_{L}$) was determined using Equation (1), where *V_obj_* is the volume of the solid cylinder, having a value of 136 ± 0.2 cm^3^. The density of the solutions was calculated with a precision of ±4 kg·m^3^.

(1) $$\rho_{L}=\frac{m_{2}-m_{1}}{V_{obj}}$$

#### 2.4.2. Surface Tension

The surface tension of the solutions was determined using a plate tensiometer K12 (Krüss). This calculation was performed following the Wilhelmy plate method, in which a thin plate of platinum placed vertically was wetted by the solution. The surface tension (*σ*) was calculated with Equation (2), where *L* corresponds to the length of the wetted plate, 𝜃 is the contact angle (which is zero in the case of a perfect wetting), and *F* is the force exerted on the vertically immersed plate. The surface tension and density of all solutions can be found in Table 1.

(2) $$\sigma =\frac{F}{L\cdot \cos\theta }$$

#### 2.4.3. Viscosity

Since process shear rates in microchannels might attain values of around 10^5^ s^−1^ [23], different techniques have to be implemented in order to cover the range of shear encountered while processing the fluids. A complete description of the methodology followed to measure and model the viscosity in this very large range of shear rates can be found in [24]. Two devices were presently used: a rotational rheometer and a microfluidic one.

Using a rotational rheometer AR1000 (TA instruments), flow tests were performed based on controlled-stress procedures in the range [6 × 10^−3^–40] Pa at 20 °C for 5 min. The geometry employed was a cone-plate with a diameter of 40 mm and an angle of 4° for the xanthan gum-based solutions. For the Newtonian solutions, a cone-plate 60 mm/2° was used instead. Due to important centrifugal forces at high shear rates, the reliability of the measurements provided by rotational rheometers was affected for shear rates higher than 10^3^ s^−1^ [24], A microfluidic rheometer (FLUIDICAM RHEO^TM^ from Formulaction, France) was then employed to measure viscosities at shear rates higher than 10^3^ s^−1^.

The principle of the measurement of this microfluidic rheometer consists of the use of laminar co-flow through a microfluidic device using two fluids: the studied fluid of unknown viscosity and a reference fluid of known viscosity. A polyethylene glycol (PEG) solution with a viscosity of 6.87 mPa·s at 20 °C was used here as the reference fluid. A microfluidic device with a 2.2 mm-wide and 150 µm-deep channel was used for these characterizations at shear rates higher than 10^3^ s^−1^. The viscosity of the unknown fluid (in this case one of our model solutions) could be determined using Equation (3). *W* corresponds to the width occupied by the fluid, which is measured by image analysis on a picture of the microfluidic ship, *Q* is the flowrate, *µ* the viscosity and the subscript *R* stands for “reference” in allusion to the reference fluid. A more detailed description of this technique can be found in [24]

(3) $$\frac{W}{W_{R}}=\frac{\mu }{\mu_{R}}\frac{Q}{Q_{R}}$$

Finally, the results obtained with the rotational rheometer and those from the microfluidic rheometer were associated and modeled using the Carreau–Yasuda model (Equation (4)). $\mu_{0}$ stands for the zero-shear viscosity, $\mu_{\infty }$ corresponds to the infinite-shear viscosity, λ represents a time constant related to the change from the 1st Newtonian plateau to the shear thinning region, *n* is a constant related to the flow index and a is a parameter defining the curvature of the transition between the 1st Newtonian plateau and the shear thinning region.

(4) $$\frac{\mu_{L}-\mu_{0}}{\mu_{0}-\mu_{\infty }}=\frac{1}{{\left(1+{\left(\lambda \dot{\gamma }\right)}^{a}\right)}^{\frac{n}{a}}}$$

### 2.5. Foaming Process

A bubble train was produced in the microchannels by injecting the gas into the liquid main flow according to the method described in Section 2.2. The flow interaction between these two fluids led to the formation of the bubble train at the intersection of the channels inside the microsystem. As a recall, the gas injection (G) was made through two channels facing each other and forming an angle of 90° with respect to the solution inlet channel (L), as presented in Figure 2. The train of bubbles then continues to evolve within the microsystem and throughout the installation, undergoing coalescence-breakup balance and resulting in an aerated matrix (foam) at the outlet of the processing line. The gas used for this study was nitrogen gas N_2_, preferable because of its chemical inertia and low solubility in water [9], which minimized the gas diffusion and thus the erosion of the small bubbles by Ostwald ripening. The flow rates used for the injection of the continuous liquid phase were in the range of 3–19 L·h^−1^ (~50–350 mL·min^−1^). These flowrates were much higher than those generally encountered in conventional microfluidics, which rarely exceed 1 mL·min^−1^ [16,17,18]. The gas flow rate was adjusted according to the desired void fraction in the final product, which was set at 0.57 ± 0.02 for this study. This value is in the range of the void fractions encountered in food foams. It corresponds to a wet foam and was selected as being compatible to the development of a dispersed flow regime in the microchannels, given the involved flow rates. This means that flow charts were established beforehand for this purpose.

### 2.6. Two-Phase Flow Characterization Inside Microchannels

#### 2.6.1. High-Speed Imaging

A high-speed camera High-SpeedStar 6 (La Vision) and a set of attachments from Navitar (0.67 adapter tube, extension tubes 10, 20 and 40 mm, 0.5X Precise Eye lens) were employed to visualize the two-phase flow inside the microchannels. As a result of the high flowrates employed, a shutter speed (exposure time) of 1 µs and a frequency of 5000 images per second (some experiments performed at 40,000 images/s) were set as the acquisition conditions for image capturing. To sufficiently illuminate the visualization area at the extremely short exposure time selected, a halogen lamp OSRAM 12 V 50 W 24° 870 lm 3000 K was used as the backlight. Image acquisition and recovery were performed using the software Photron FASTCAM Viewer. Then, the images were processed and analyzed (feature extraction) using the open-source software ImageJ.

#### 2.6.2. Bubble Size Distribution

For each trial, around 50 to 100 processed images were needed to obtain a significant number of bubbles (higher than 500) for the analysis of size distributions. Since some bubbles may appear in several successive photos, one image out of every four consecutive frames was selected in order to avoid double estimations of the same bubbles. The feature extraction from ImageJ provided the maximum axial dimension *L_b_* (maximum caliper diameter) as well as the maximum radial dimension *B_b_* (minimum caliper diameter), which correspond to the largest and smallest dimensions, respectively, of the bubble as if they were measured using a caliper. The volume of a bubble (*V*) was estimated using Equation (5). An equivalent spherical diameter (*d_eq_*) was found using Equation (6). Then, Boyle’s law (Equation (7)) allowed us to calculate the diameters at atmospheric pressure (*d_atm_*). Finally, the diameters at atmospheric pressure were used for the calculation of number and volume size distributions. Other relevant parameters for the study of dispersed systems were also determined, such as the Sauter diameter, d_32_ [25,26,27,28]. The uncertainty regarding the bubble size and shape due to the selected image acquisition conditions was found to be around 5.7% [21], which is sufficiently low to ensure reliability.

(5) $$V=\frac{\pi \;B_{b}{}^{2}L_{b}}{4}$$

(6) $$V=\frac{\pi \;d_{eq}^{3}}{6}$$

(7) deqdatm=(patmpinj+patm)13

#### 2.6.3. Characterization of Hydrodynamic Conditions

Characterizations of the hydrodynamic conditions were carried out in order to identify several parameters such as superficial fluid velocities, pressure drops and void fractions which are essential for the understanding of the physical phenomena taking place. The superficial velocities *U_L_* and *U_G_* were calculated knowing the liquid and gas volume flow rate over the cross section of the microchannel. Since the foams were produced in the dispersed regime, i.e., in the absence of the formation of a plug of gas in the whole section of the microchannel, the void fraction at different stages of the process was determined using Equation (8). Similarly, the apparent shear rate (${\dot{\gamma }}_{app}$) was estimated using Equation (9), where *K_s_* is a geometric constant having a value of 7.11 for square channels [29]. The hydraulic diameter, *D_h_*, is presently the square side. The apparent viscosity of the liquid phase (*μ_L_*) was calculated using the Carreau–Yasuda model (Equation (4)).

(8) $$\alpha =\frac{U_{G}}{U_{G}+U_{L}}$$

(9) $${\dot{\gamma }}_{app}=k_{S}\frac{U_{L}}{D_{h}}$$

The pressure sensors in Figure 1 allowed the measurement of the pressure loss (Δ*p*_1_) in the microsystem. The downstream pressure loss (10 and 12 in Figure 1) was roughly estimated thanks to the Hagen–Poiseuille law (Equation (10)), where *L* is the tube length and *D* is the tube diameter. Knowing that this equation is applicable for laminar flows of Newtonian fluids, which was not the case here, the objective was to complete in a basic way the pressure profile, i.e., in intermediate areas of the process line where no pressure probe was available.

(10) $$\Delta p=\frac{32\mu_{L}U_{L}L}{D^{2}}$$

**Figure 3:**
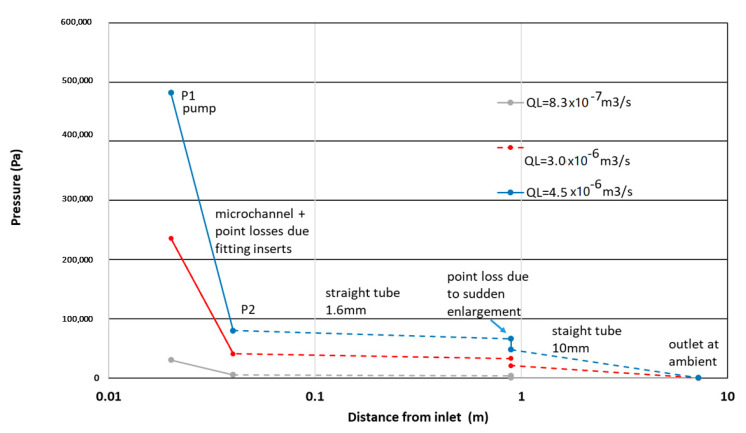
Example of the profile of the relative pressure along the microchannel hydraulic loop for the WPI3XG04 solution and three different flowrates. The log scale presentation of the axial coordinate aims to show more clearly the important weight of the microchannel in the global pressure drop. The downstream pressure drop is shown as a dotted line, and it concerns the successive tubes of the relaxation line and is roughly evaluated.

An example of the experimental pressure profile is given in Figure 3. It was obtained with a combination of measured and evaluated pressures. Evaluated pressures result from the computation of the Poiseuille law (Equation (10)) in the two long straight tubes considering a monophasic flow of WPI3XG04 solutions. This type of profile, available for all the trials, is useful to illustrate the pressure to be overcome and is also useful to discuss its effect on the bubble diameter.

Finally, the interactions of the different forces involved in the formation, deformation and breakup of interfaces were evaluated using dimensionless numbers: the Reynolds number, *Re* (Equation (11)), the capillary number, *Ca* (Equation (12)), and the Weber number, *We* (Equation (13)). These numbers were expressed with respect to the fluid that contributed the most to the flow characteristics [30,31], which in the case of foams corresponded to the liquid phase, and hence the subscript *L* in these equations. In Equations (11)–(13), $\rho_{L}$ corresponds to the liquid density and σ is the surface tension.

(11) $$Re_{L}=\frac{U_{L}\rho_{L}D_{h}}{\mu_{L}}$$

(12) $$Ca_{L}=\frac{\mu_{L}U_{L}}{\sigma }$$

(13) $$We_{L}=\frac{\rho_{L}U_{L}^{2}D_{h}}{\sigma }$$

Depending on the Reynolds number, either the viscous forces dominate, in which case the capillary number is the relevant parameter, or the inertial ones dominates, and then the Weber number is appropriate. In the transitional situations, both numbers can be useful to account for the same magnitude of laminar and inertial forces.

### 2.7. Foam Characterizations

#### 2.7.1. Foam Rheology

A controlled-stress rotational rheometer AR1000 (TA Instruments) with a parallel plate geometry was employed for the rheological characterizations of produced foams. The parallel plates were covered with waterproof sandpaper (grain size 60 µm) to prevent wall slip. The gap was set at 2000 µm (about 10 times the bubble mean size). Dynamic and flow tests were performed as follows:

##### Dynamic Test

Frequency sweeps were carried out to measure the mechanical moduli (G’ and G’’) and thus to characterize the viscoelastic properties of the foams produced. Measurements were performed at a temperature of 20 °C in the frequency range 5–0.5 Hz. The sweep was carried out in descending order, since low frequencies imply longer times that might alter foam structure. A strain percentage of 1% (linear viscoelastic domain) was used for these tests. Measurements were performed in triplicate.

##### Flow Test

Flow tests were performed to characterize the foam viscosity. These were conducted at 20 °C in the stress range of 0.01–50 Pa with a duration of 4 min for the ramp step up. Measurements were performed in triplicate.

#### 2.7.2. Bubble Size Distribution

For the characterization of the bubble sizes, the foam was trapped in a visualization cell located at the end of the processing line (number 13 in Figure 1). Then, images of the foam structure were obtained using a camera Sony CCD XCL-5005 with a resolution of 5 M pixels and the set of optic attachments from Navitar described in Section 2.6.1. The image capture and recovery were performed using a LabVIEW software (National Instruments, Austin, TX, USA).

The images were processed and analyzed using the open-source software ImageJ. For feature extraction, around 10 photos for each trial were enough to ensure a significant number of analyzed bubbles (>500). With the different elements and conditions used for image acquisition, the minimum size of the bubbles that was possible to adequately characterize was of the order of 30 to 40 µm. Bubble sizes were determined with the same procedure described in Section 2.6.2 without the use of Boyle’s law, since the bubbles of the foams were already at atmospheric pressure.

#### 2.7.3. Stability

For the characterization of their stability, samples of foams were collected in 100 mL graduated cylinders and the initial height was measured (H_0_). Then, the evolution of the foam height (H) was monitored over time and registered as a normalized height (H/H_0_). The stability was determined as a function of the drainage half-life time (t_1/2_) that corresponds to the time after which the foam had lost 50% of its volume. The measurements were performed in triplicate.

## 3. Results and Discussions

### 3.1. Bubble Train in the Micro-Channel

This paragraph describes the flow typology and the mechanisms of bubble formation and breakup in the microchannels with WPI/XG solutions at high flow rates. As some of these experiments were already analyzed in a previous study [21], only a brief description of the results, useful for the following discussions, will be provided in this section.

#### 3.1.1. Flow Typology and Mechanism of Bubble Breakup in the Microchannels

**Figure 4:**
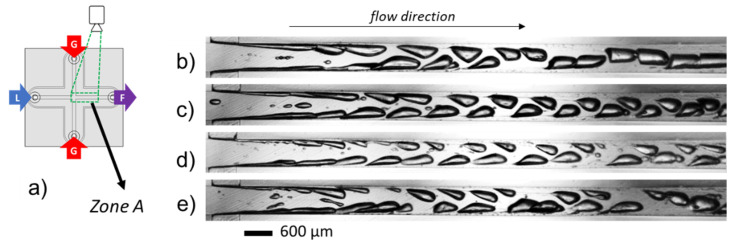
(**a**) Visualization at the area of bubble formation (Zone A) in the channel 600 × 600 µm^2^ of the device CX600. (**b**–**e**) Images at different process conditions: (**b**) Q_vL_ = 11 L·h^−1^ − Q_mG_ = 21.6 g·h^−1^. (**c**) Q_vL_ = 13.7 L·h^−1^ − Q_mG_ = 26.5 g·h^−1^. (**d**) Q_vL_ = 16.4 L·h^−1^ − Q_mG_ = 31 g·h^−1^. (**e**) Q_vL_ = 19 L·h^−1^ − Q_mG_ = 36.4 g·h^−1^; liquid phase: model solution WPI3XG04, gas phase N_2_. Figure reproduced from [22] with permission from Elsevier.

The first event of gas–liquid interfacial breakup takes place at the level of the gas injection due to the Kelvin–Helmholtz hydrodynamic instabilities. This breakup of the gaseous fluid vein is an essential means to obtain individual bubbles. Within the operated conditions, it allows for obtaining a train of bubbles with similar volumes. Each bubble is formed near the wall and presents a teardrop shape characteristic of bubbles flowing through a shear-thinning fluid. Then, bubbles gradually migrate along the outlet channel and towards its center, where they undergo lower shear. Figure 4b–e present the effect of flow rates from 11 to 19 L·h^−1^ on the bubble train in the mixing microchannel. These flow rates correspond to estimated Reynolds numbers in the range of 1900 to 3700. In such conditions, the estimated bubble average velocities vary from 14 to 20 m·s^−1^ in this zone (Zone A). No turbulence is observed in the two-phase flow, despite Reynolds numbers reaching rather high values (Table 2). This could be explained by the role of the gas bubbles acting as solid walls. Thus, the hydraulic diameter effectively encountered by the liquid solution is lower. The use of an interstitial Reynolds number could be more appropriate, but it is difficult to evaluate it, knowing that we only have two-dimensional information on the flow. Another factor that might contribute to the ordered flow despite high Reynolds numbers would be the shear-thinning properties of xanthan gum.

As can be seen from Figure 4b–e, the size of bubbles progressively decreases with increasing flowrates. A longer distance is needed for the bubbles to reach the center of the channel, which is the result of greater inertial forces involved in the microchannel.

**Figure 5:**
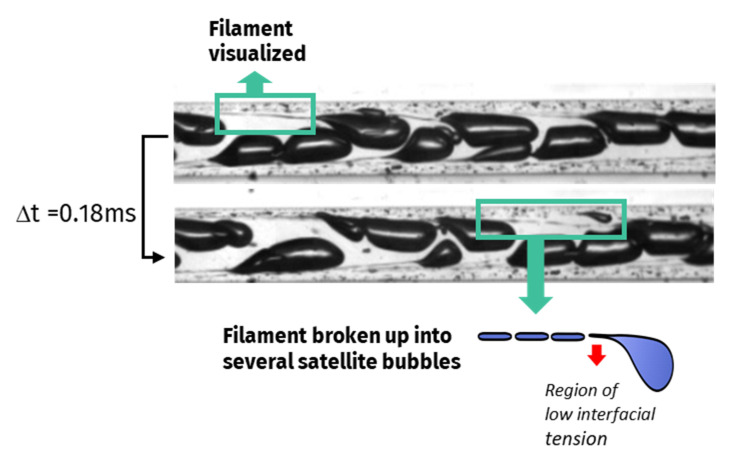
Tip-streaming bubble breakup mechanism identified in the mixing channel of all devices. Visualizations with the device CX600 at a liquid flowrate of 11 L/h. Liquid phase: model solution WPI3XG04. Gas phase: N_2_. At the bottom left: enlarged view of a bubble and its schematic representation. In the photos, a filament (circled in green) is shown before and after its break-up into satellite droplets; an interval of 0.18 s had elapsed between the two pictures.

**Figure 6:**
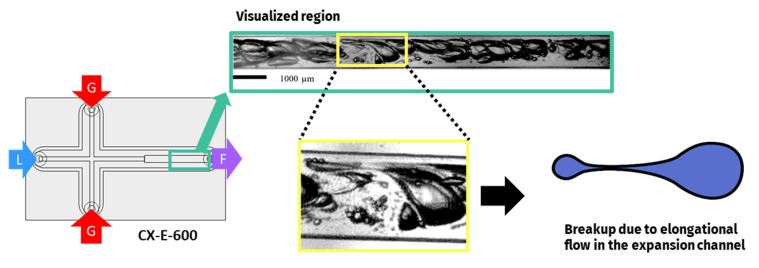
Bubble binary breakup mechanisms identified for the two-phase flow in the expansion channel. Visualizations from device CX-E-600 at a liquid flowrate of 3 L/h. Liquid phase: model solution WPI3XG04. Gas phase: N2. Adapted from [22] with permission from Elsevier.

After their formation, the bubbles flowing through the microchannels present two additional breakup mechanisms: tip streaming (Figure 5) and binary breakup (Figure 6).

Tip-streaming can be identified in Figure 5 by the formation of gaseous filaments behind the bubbles travelling near the walls, where viscous shear stresses are stronger. Interfacial tension gradients are the main cause leading to this type of breakup mechanism [30,32,33,34]. It originates from a deficit of whey proteins at the interface of the bubbles, concomitant to the curvature inversion of the interface. To support this view, the estimated protein adsorption time was compared to the lifetime of the visualized bubble (time of formation + residence time in the microchannel). For β-lactoglobulin, the predominant molecule in whey protein isolates, the protein adsorption time was estimated at 2 ms. It is found to be greater than the lifetime of the bubbles (time of formation < 0.2 ms + residence time < 1.3 ms for the conditions in Figure 4) calculated through image analysis. A more detailed procedure of this calculation can be found in [21]. As a result, under the studied conditions, the gas–liquid interfaces were not fully covered, resulting in the establishment of interfacial tension gradients and subsequent tip streaming phenomenon.

A second major breakup mechanism is identified in the expansion channel of CX-E-600, represented in Figure 6.

The expansion channel is studied here, as it is supposed to mimic the transitions towards sections with a larger diameter that occur in industrial processing lines in particular, but not exclusively, in the relaxation line. In the present process line studied, a first enlargement occurs in the outlet port of the microsystem, as mentioned in Section 2.2. Unfortunately, technically speaking, the expansion of the surface area of the cross section of the microchannel implies a superposition of an important number of bubbles. This makes the liquid vein too thick to allow a clear visualization of the bubble train in the conditions of flowrates previously studied (11–19 L/h). Therefore, the studied liquid flowrates are limited to around 3 L·h^−1^ (50 mL·min^−1^) to characterize the behavior of individual bubbles in this area. Under these conditions, the two-phase flow in the expansion is laminar, with a liquid Reynolds number of around 110 (Table 3). However, due to the sudden expansion, the flow is not fully developed, which gives rise to recirculation and backflow phenomena (secondary flows), leading to elongated bubbles. Some of the visualized elongated bubbles recover their shape while others split in two, hence the name of binary breakup given to this mechanism. Figure 6 illustrates the elongation and binary breakup of a bubble in the expansion.

In the next section, the structure of the foam obtained at the outlet of the processing line is characterized. The size distribution of the bubbles in the foam is also compared to that inside the mixing microchannel.

#### 3.1.2. Bubble Evolution in the Experimental Processing Line

**Figure 7:**
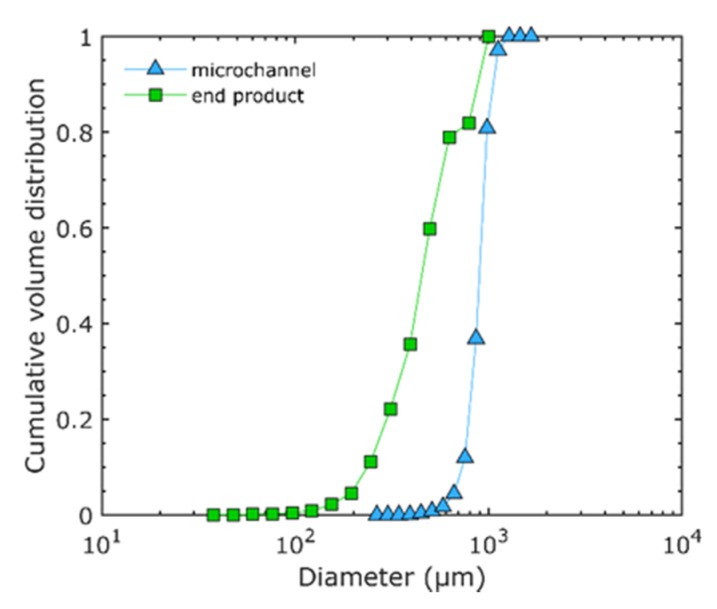
Cumulative volume distribution of bubbles using the model solution WPI3XG04 and the microsystem CX600 at Q_vL_= 11 L·h^−1^, Q_mG_ = 21.6 g·h^−1^, P_inj_ = 3.83 bar. Size distribution of bubbles inside the microfluidic device (triangles) and in the end product (squares). The diameters of bubbles inside the microchannels are normalized to the atmospheric pressure (outlet pressure). Adapted from [22] with permission from Elsevier.

The bubbles produced in the microchannels are dimensionally different from those observed in the foam obtained at the end of the experimental processing line. Figure 7 allows one to compare the size distribution of the bubbles formed in the mixing microchannel (triangles) to that of those in the final foam (squares) at a liquid flowrate of 11 L·h^−1^. For the sake of this comparison, the size of the bubbles measured in the microchannel is normalized, i.e., recalculated in the conditions of the atmospheric pressure (outlet of process line). It is observed that the size distribution of the bubbles in the foam is shifted towards smaller diameters compared to the size distribution of bubbles in the microchannel, i.e., “in line”. This confirms that a size evolution (governed by breakup phenomena) is taking place along the processing line. It is also noteworthy that the bubble sizes are more homogeneous in the microchannels than in the foam, i.e., at the exit of the process line. This is shown by a steeper slope or lower span (D_90_-D_10_/D_50_ = 0.34) for the bubble distribution inside the microchannel when compared to the bubble distribution in the foam (span = 1.45). The less homogeneous size distribution observed in the outlet of the process is explained by the presence of multiple types of breakup events in the process line. The void fraction is also modified, changing from 0.26 in the mixing microchannel to 0.57 in the final foam.

The question that then arises is whether there is an impact of the channel geometry (abrupt expansion) on the structural and usage properties of the foams produced. To answer this, a comparison of the properties of the foams was carried out. In this objective, the impact of varying liquid flowrate and XG concentration was investigated for the foams produced with the device CX-E-600.

### 3.2. Impact of Processing Conditions on Foam Functional Properties

The usage properties of foams, namely their texture and stability, are related to their structural characteristics, i.e., the void fraction, the bubble size and the size distribution. For this study, all the foams were produced at a fixed void fraction of 0.57 ± 0.02. They were wet foams, the bubbles of which were not structured in a close packing configuration. Additionally, since the WPI concentration remained equal to 3% (no variation of the interfacial tension), all the eventual changes in the usage properties were a direct consequence of a structure modification (in terms of bubble size) and then of the rheological properties of the liquid phase governed by the xanthan gum.

#### 3.2.1. Effect of the Device Geometry

**Figure 8:**
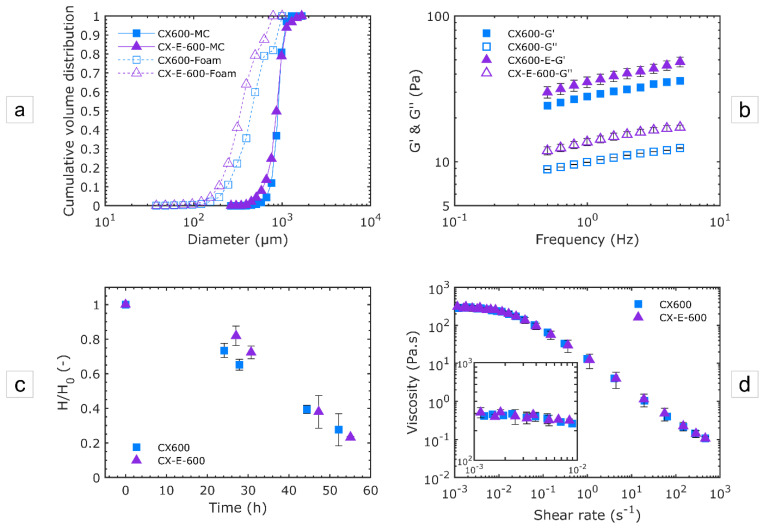
Effect of the expansion channel (CX-E-600 vs. CX600) on the structural and end-used properties of foams produced with the model solution WPI3XG04 at Q_vL_= 11 L·h^−1^ and Q_mG_ = 21.6 g·h^−1^. (**a**) Bubble size distributions in cumulative volume in the mixing channel (MC) and in the end product (foam). The diameters of bubbles inside the microchannels are normalized to the atmospheric pressure. (**b**) Mechanical moduli of foams at 20 °C. (**c**) Stability of foams. (**d**) Viscosity characterization of foams at 20 °C.

Figure 8 summarizes the results for the structural (size distribution) and usage (rheological and stability) properties of foams produced using the devices CX600 and CX-E-600 with the solution WPI3XG04 at a liquid flowrate of 11 L·h^−1^.

Figure 8a presents the size distribution at two locations: in the mixing channel (filled markers and solid lines) and in the foam obtained at the outlet of the processing line (open markers and dashed lines), respectively. Similar bubble sizes and size distributions are observed in the mixing channel of the two devices (span of 0.34 for CX600 and 0.46 for CX-E-600). However, bubbles measured in the foams (open markers) tend to be smaller within the foam produced with CX-E-600, despites being similarly distributed to those in foams produced with CX600 (i.e., similar span value of 1.45 for foams produced with CX600 and CX-E-600). The smaller bubbles in the foams produced with CX-E-600 are due to an intensification of the gas–liquid interface breakup inside the expansion channel, as explained in the previous section. This difference in structure has an important effect on the mechanical moduli of the foams (Figure 8b); the elastic modulus (G’) is indeed higher for the foams produced with CX-E-600. This means that the foams are firmer, with slightly improved stability (Figure 8c). Finally, no significant difference was observed concerning the viscosity of the foams (Figure 8d). One has to keep in mind that the foam produced at the outlet presents a relatively high liquid ratio (void fraction~0.57) compared to dry foams. Therefore, the rheological properties of the liquid phase are predominant over the foam structure on the viscosity of the foams presented in Figure 8d.

#### 3.2.2. Effect of the Flowrate

**Figure 9:**
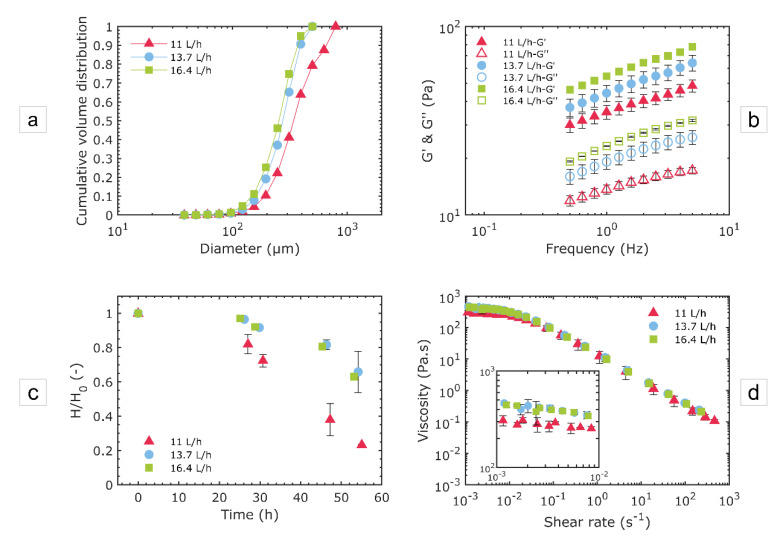
Effect of the liquid flowrate on the structural and usage properties of foams produced with the device CX-E-600 and the model solution WPI3XG04 at various conditions: Q_vL_ = 11 L·h^−1^ − Q_mG_ = 21.6 g·h^−1^, Q_vL_ = 13.7 L·h^−1^ − Q_mG_ = 23 g·h^−1^ and Q_vL_ = 16.4 L·h^−1^ − Q_mG_ = 31.0 g·h^−1^. (**a**) Bubble size distributions in the foams in cumulative volume. The diameters of bubbles inside the microchannels are normalized to the atmospheric pressure. (**b**) Mechanical moduli of foams at 20 °C. (**c**) Stability of foams. (**d**) Viscosity characterization of foams at 20 °C.

The effect of the flowrate on the foam properties was investigated for the foams produced with the device CX-E-600 and with the model solution WPI3XG04 (Figure 9).

First, a trend towards smaller bubbles and narrower distributions can be identified with an increasing liquid flowrate (Figure 9a); with span values of 1.45, 0.85 and 0.96 for 11, 13.7 and 16.4 L·h^−1^, respectively. Greater inertial forces are present at higher flowrates, which explains this trend. This intensified breakup, with an increasing flowrate, in turn has an impact on the firmness of foams. Foams produced at higher flowrates indeed present more structured networks, as revealed by the larger values of the elastic modules (Figure 9b). In terms of stability (Figure 9c), clear differences were identified between 11 L·h^−1^ and higher flowrates (13.7 and 16.4 L·h^−1^). However, no significant difference was found between the two highest liquid flowrates employed (13.7 and 16.4 L·h^−1^). Similar stabilities between 13.7 and 16.4 L·h^−1^ could be explained by similar size distributions observed in Figure 9a. Slight differences in the viscosity characterizations were identified as well (Figure 9d). This is in alignment with the hypothesis put forward in Section 3.3.1; that is, the predominant role of liquid rheology in the viscosity of foams with a rather low void fraction (0.57).

#### 3.2.3. Effect of Xanthan Concentration

**Figure 10:**
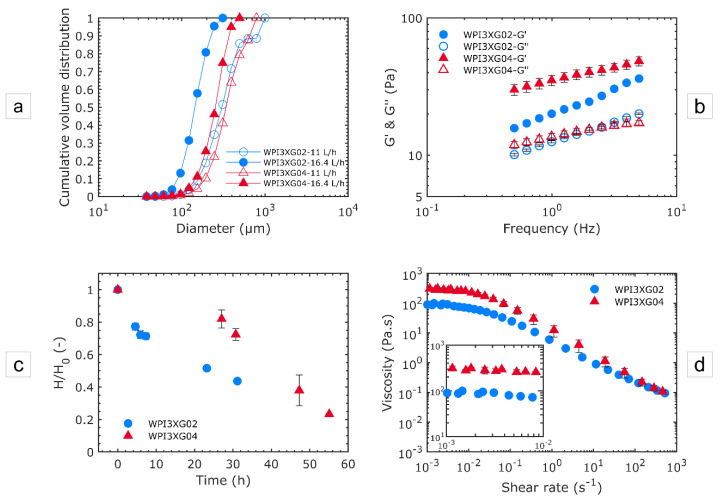
Effect of xanthan gum concentration on the structural and usage properties of foams produced with the device CX-E-600 and the model solutions WPI3XG04 and WPI3XG02. Circles stand for WPI3XG02 and triangles for WPI3XG04. Empty symbols stand for 11 L·h^−1^ and full symbols for 16.4 L·h^−1^. (**a**) Bubble size distributions in the foam in cumulative volume at two flowrate conditions: Q_vL_ = 11 L·h^−1^ − Q_mG_ = 21.6 g·h^−1^ and Q_vL_ = 16.4 L·h^−1^ − Q_mG_ = 31 g·h^−1^. The diameters of bubbles inside the microchannels are normalized to the atmospheric pressure. (**b**) Mechanical moduli of foams at 20 °C for Q_vL_ = 11 L·h^−1^ − Q_mG_ = 21.6 g·h^−1^. (**c**) Stability of foams for Q_vL_ = 11 L·h^−1^ − Q_mG_ = 21.6 g·h^−1^. (**d**) Viscosity characterization of foams at 20 °C for Q_vL_ = 11 L·h^−1^ − Q_mG_ = 21.6 g·h^−1^.

The effect of two xanthan concentrations, 0.4 and 0.2% *w*/*w* (respectively designated as WPI3XG04 and WPI3XG02), on the foam properties was investigated using the device CX-E-600 (Figure 10). In Figure 10a, the effect of XG concentration on the bubble sizes in the foams is evaluated for two liquid flowrates. For a same value of flowrate, the bubble sizes shift towards smaller values when the concentration decreases from 0.4 (triangles) to 0.2% (circles). Conversely, size distributions become more heterogeneous (higher span) when the concentration is reduced; for 11 L·h^−1^, the span is 1.45 for the solution WPI3XG04 and 1.55 for WPI3XG02, and for 16.4 L·h^−1^, it is 0.96 for WPI3XG04 and 1.04 for WPI3XG02. This trend is due to higher inertial forces applied to the bubbles during their genesis and travelling through experimental processing line. The decrease in bubble size with a lower XG concentration is all the more remarkable at the highest the flowrate (16.4 L·h^−1^). In contrast to the other previously analyzed factors (design of microsystem and flow rate), foam firmness (Figure 10b) and stability (Figure 10c) did not vary inversely with bubble size (Figure 10a). They were enhanced at XG 0.4%, showing that the effect of viscosity predominates over that of the structure. Finally, flow tests revealed a considerable difference in viscosity for the foams produced with the two XG concentrations (Figure 10d). Foams with a more viscous liquid phase displayed higher viscosity values. This demonstrates the large impact of the XG concentration on the stabilization of the foam networks.

In conclusion, the structural and textural characteristics of the foams produced are strongly affected by experimental processing conditions such as the design of the device, the value of flowrates employed and the concentration of xanthan gum.

For the same formulation, the end-use properties of foams (firmness and stability) result from their structure—that is, driven by the size distribution of the bubbles formed in the microchannel. At a given xanthan concentration, increasing the flowrate allows for obtaining a smaller average bubble size, which logically reinforces the stability and firmness of the foam. On the other hand, increasing the xanthan concentration is another means to attain higher stability and firmness, despite larger bubble sizes being visualized in the foam compared to the case of a smaller xanthan concentration.

Foams obtained with the highest tested xanthan concentration and flowrate are more stable in the short term (<30 h), but in the long term (up to 55 h), the trend of evolution of the stability joins that of the foams obtained with the lower concentration tested. This means that the benefit brought about by a higher concentration of xanthan is lost over time, as the granulometry is less favorable for the long-term stability of the foam.

The rheological behavior of the liquid phase at the process shear rate therefore also plays a major role in the establishment of the breakup phenomena and subsequent structuring of the end product. This is an aspect that we will now develop by investigating both the surface-active and rheological properties of the solutions, through different formulations.

### 3.3. Effect of the Formulation on the Typology of the Bubble Train in the Microfluidic Device

To understand the respective role of the biosourced molecules selected on the two-phase flow phenomena visualized inside the microchannels, solutions containing only one of the biopolymers are prepared. Thus, one of the solutions is formulated using only xanthan gum at 0.4% *w*/*w* (XG04), while another solution consists only of whey protein isolates at 3% *w*/*w* (WPI3). Visualizations are carried out on two sections at different flowrates. The mixing channel of the device CX600 is observed at Q_vL_ = 11 L·h^−1^. The expansion channel of the device CX-E-600 is visualized at Q_vL_ = 3 L·h^−1^, as it is recalled that its larger section prevents obtaining clear visualizations of the bubbles at higher flow rates. The estimated apparent shear rates at these two locations are around 10^5^ s^−1^ and 5 × 10^3^ s^−1^, respectively.

Additionally, a comparison is proposed with a solution based on glycerol at 50% *w*/*w* so as to investigate a Newtonian fluid, i.e., without shear thinning characteristics, for which the viscosity is close (slightly higher) to the apparent viscosity of the xanthan solution at the microchannel shear rates. However, the properties of the latter foams were not measured, since they did not yield stable foams.

#### 3.3.1. Role of the Whey Proteins

**Figure 11:**
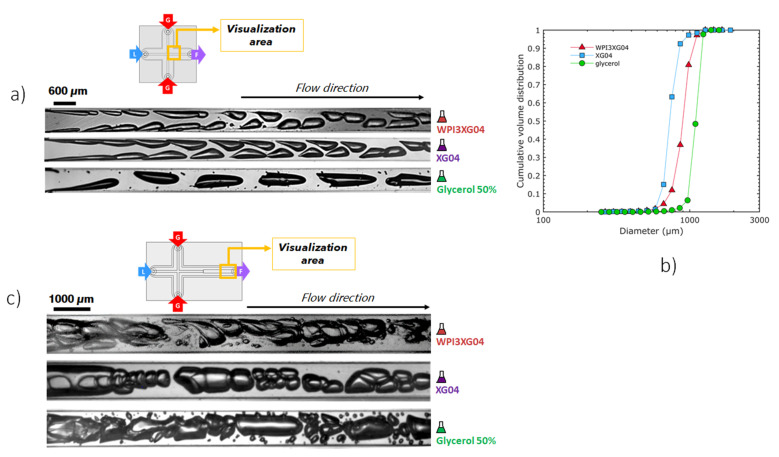
Effect of formulation on two-phase flow typology and bubbles sizes. (**a**) High-speed visualizations of the two-phase flow inside the mixing channel of the device **CX600** for solutions WPI3XG04, XG04 and glycerol at Q_vL_ = 11 L·h^−1^ and Q_mG_ = 21.6 g·h^−1^. (**b**) Bubble size distribution in cumulative volume for bubbles inside the mixing channel of **CX600** for solutions WPI3XG04, XG04 and glycerol at Q_vL_= 11 L·h^−1^ and Q_mG_ = 21.6 g·h^−1^. The diameters of bubbles inside the microchannels are normalized to the atmospheric pressure. (**c**) High-speed visualizations of the two-phase flow inside the expansion channel of the device **CX-E-600** for solutions WPI3XG04, XG04 and glycerol at Q_vL_ = 3 L·h^−1^ and Q_mG_ = 5 g·h^−1^.

The flow typology observed with the reference solution, WPI3XG4, is compared to that with a protein-free xanthan solution (XG04) to identify the role played by the whey proteins on the flow phenomena. Concerning the solution XG04 (Figure 11a), the bubbles formed in the mixing channel maintain the characteristic teardrop shape. However, they do not present the formation of thin gaseous filaments at the tips of the bubbles; thus, the tip-streaming breakup mechanism is not identified. This confirms the necessity of surface-active molecules to observe this phenomenon. These two solutions were both non-Newtonian with similar values of apparent viscosity, 2.1 × 10^−3^ Pa·s for XG04 and 2.5 × 10^−3^ Pa·s for WPI3XG04 (Table 4); this could explain why the shapes of bubbles for these two solutions are similar.

**Figure 12:**
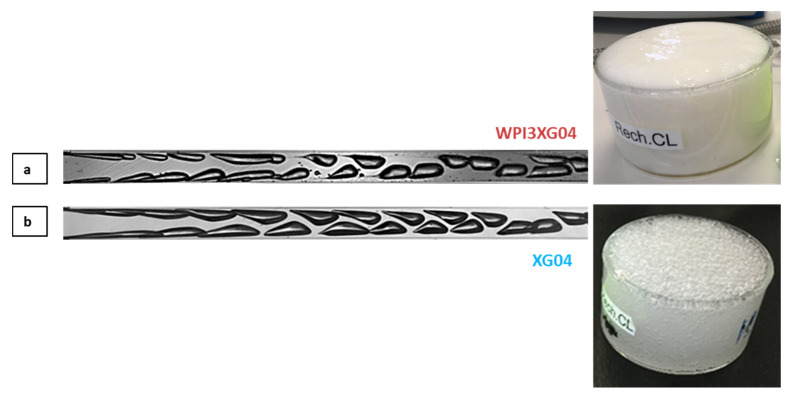
Visualizations of bubbles in the outlet microchannel and visual aspect of foams obtained at the end of the process when using the device CX600 and flowrates *Q_vL_* = 11 L·h^−1^ and *Q_mG_* = 21.6 g·h^−1^. (**a**) Solution WPI3XG04. (**b**) Solution XG04. The crystallizing dishes have an approximative diameter of 8 cm and a height of about 5 cm.

On the other hand, at the same flow rate conditions, bubbles travelling through the glycerol solution, which has a Newtonian behavior and a higher apparent viscosity under the process conditions (6 × 10^−3^ Pa·s), present bullet shapes, which is rather typical in laminar flows. The bubbles also reach the center of the channel at a much shorter distance from the genesis point. The slightly higher viscosity at the process shear rate and lower inertial forces in this case (Table 4) lead to larger bubbles for glycerol compared to WPI3XG4 and XG4 (Figure 11a,b). On the other hand, a0 similar tendency towards a decrease in bubble size was observed with an increase in the Reynolds number, at 950, 1980 and 2379 for glycerol, WPI3XG04, and XG04, respectively (Table 4). Despite the high monodispersity observed for the three populations (span of 0.34 for WPI3XG04, 0.11 for glycerol and 0.35 for XG04), no foamed product is obtained at the outlet of the process for XG04 and glycerol (Figure 12). Indeed, the various successive increases in the hydraulic diameter along the process line allow a rather smooth transition towards atmospheric pressure, but the simultaneous velocity decrease of the diphasic flow enhances coalescence in the case of liquids free of surfactant. These findings confirm that it is not enough to study the ability of microchannels to form bubbles when using these devices for foaming/emulsification applications and that a more rigorous approach has to be developed to select a formulation.

**Figure 13:**
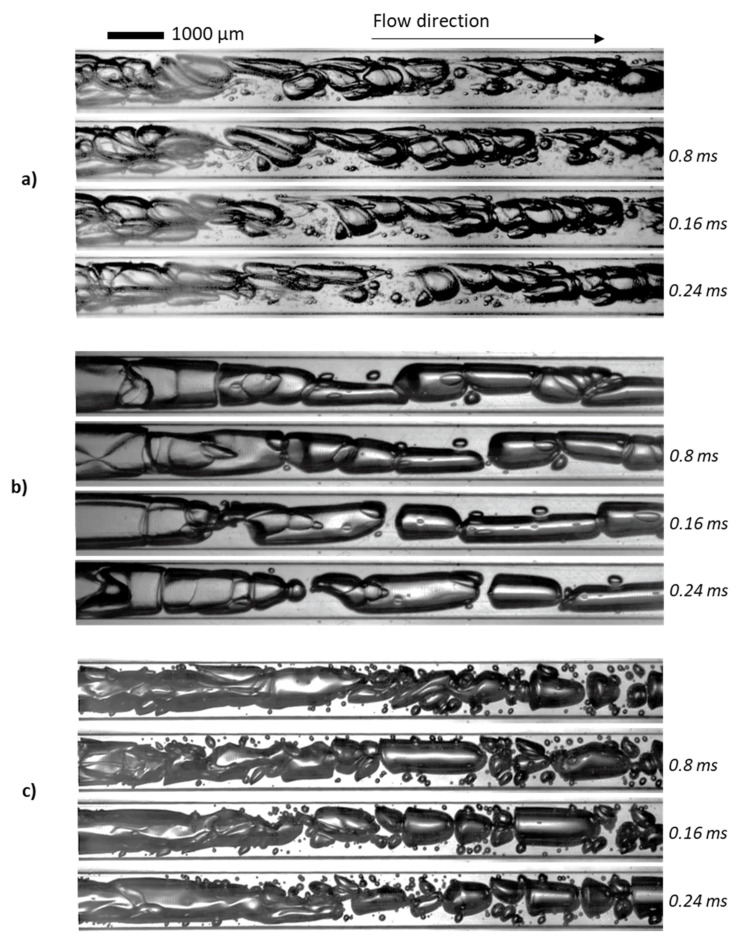
Series of high-speed images selected at an interval of 0.8 ms for the two-phase flow inside the expansion channel of the device CX-E-600. Flowrates used: *Q_vL_* = 3 L·h^−1^ et *Q_mG_* = 5 g·h^−1^. (**a**) WPI3XG04. (**b**) XG04. (**c**) Glycerol 50%. Image acquisition frequency of 5000 images per second, resulting in an interframe time of 0.2 ms.

Figure 11c shows the two-phase flow in the expansion channel of CX-E-600. Successive images of these cases are also presented in Figure 13. In this section of the device, the viscosity of the shear-thinning solutions increases due to a sudden drop in the shear rate from 10^5^ to 5.5 × 10^3^ s^−1^. Despite similar Reynolds numbers for xanthan gum-based solutions, at 110 and 162 for WPI3XG04 and XG04, respectively (Table 5), only the solution containing the proteins and the xanthan gum (WPI3XG04) displays bubble binary breakup by elongation and splitting of the interface. Conversely, bubbles travelling through the solution with xanthan gum only (XG04) tend to coalesce. With the glycerol solution, the flow seems slightly more chaotic, despite the Reynolds number being similar to that of the other two solutions. Numerous tiny bubbles can be observed with the glycerol solution, and therefore it has a more polydisperse bubble size distribution. The visualizations made within the expansion channel (Figure 11c and Figure 13) along with those performed in the mixing channel (Figure 11a) demonstrate the importance of the shear-thinning properties of xanthan gum for controlling bubble shapes and sizes even at high shear rates in the range of 10^3^ to 10^5^ s^−1^ encountered in microchannels.

#### 3.3.2. Role of the Xanthan Gum

**Figure 14:**
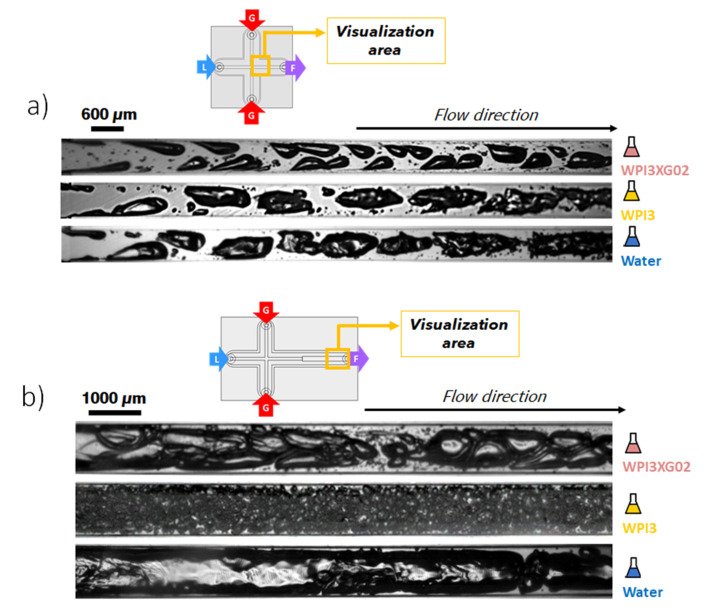
Effect of the formulation on two-phase flow typology. (**a**) High-speed visualizations of the two-phase flow inside the mixing channel of the device CX600 for the solutions WPI3XG02, WPI3 and water at Q_vL_ = 11 L·h^−1^ and Q_mG_ = 21.6 g·h^−1^. (**b**) High-speed visualizations of the two-phase flow inside the expansion channel of the device CX-E-600 for the solutions WPI3XG02, WPI3 and water at Q_vL_ = 3 L·h^−1^ and Q_mG_ = 5 g·h^−1^.

The objective is now to investigate more specifically the role of xanthan gum on the flow phenomena. For that purpose, a solution using only whey protein isolates at 3% *w*/*w* (WPI3) is employed. Figure 14 presents the two-phase flow phenomena for the solution WPI3 as well as for two other formulations (WPI3XG02 and water) inside the mixing channel of the device CX600 (Figure 14a) and inside the expansion channel of the device CX-E-600 (Figure 14b). The series of successive images for these cases are presented in Figures S1 and S2. The solution WPI3XG02 (3% proteins and 0.2% xanthan gum) is employed for comparison, since it presents a similar contribution of the inertial forces to the flow phenomena, as in the case of WPI3: the Reynolds number is 3500 and 3200 for WPI3 and WPI3XG02, respectively (Table 5). This similarity is explained by the strong shear thinning behavior of xanthan solutions leading to viscosities of 1.40 and 1.50 × 10^−3^ Pa·s at 10^5^ s^−1^ for WPI3 and WPI3XG02, respectively (Table 6). Regarding the test with tap water as the liquid phase, the purpose is to compare the two previous solutions (WPI3 and WPI3XG02) with a liquid fluid devoid of both biosourced constituents employed in the formulation of the model solutions (WPI and XG).

Depending on whether or not the solution contains xanthan gum, noticeable differences in bubble shape can be identified in the mixing channel (Figure 14a). For the protein solution and water, the bubbles exhibit similar bullet-shaped appearance identified for the glycerol solution in Section 3.3.1, but with a much more irregular shape of the interfaces, due to more intense inertial forces, as evidenced by the Reynolds numbers (cf. Table 4 and Table 6). As for the glycerol solution, the bubbles flowing through water and WPI3 tend to arrange themselves in the center of the channel. In contrast, bubbles flowing through WPI3XG02 present teardrop shapes and spiked-like arrangements similar to those described in Section 3.1.1 when using a more concentrated model solution (WPI3XG04). It is noteworthy that despite an excess concentration of proteins, the train of bubbles with WPI3 is remarkably similar to that of water. Another interesting observation is that for the WPI3XG02 solution, the bubbles do not present any roughness and surface alterations, as observed for WPI3, despite their close Reynolds numbers of 3200 and 3500, respectively (Table 6). This seems to indicate that the presence of xanthan would induce friction reduction and flow stabilization.

Additionally, the analysis of the flow at the expansion channel (Figure 14b and Table 7) provides a more interesting insight into the role of proteins and xanthan gum. Bubbles formed through WPI3 are much more fractionated than those in WPI3XG02 and water. Bubbles flowing through water actually coalesce due to the absence of surface-active molecules. In the expansion area, where the fluid velocity is reduced, these images clearly demonstrate the well-known essential role of WPI as an interfacial stabilizer. Xanthan gum, despite the presence of WPI (solution WPI3XG02), seems to limit bubble breakup. However, xanthan gum remains essential to obtain a stable foam at the exit thanks to its high viscosity at rest. It can be concluded that xanthan gum stabilizes the shape and size of bubbles from the moment of their genesis in the mixing channel, which has important implications in controlling the structural properties of foams for microfluidic technologies.

#### 3.3.3. Effect of Formulation on Bubble Characteristics in the CX600 Device

**Figure 15:**
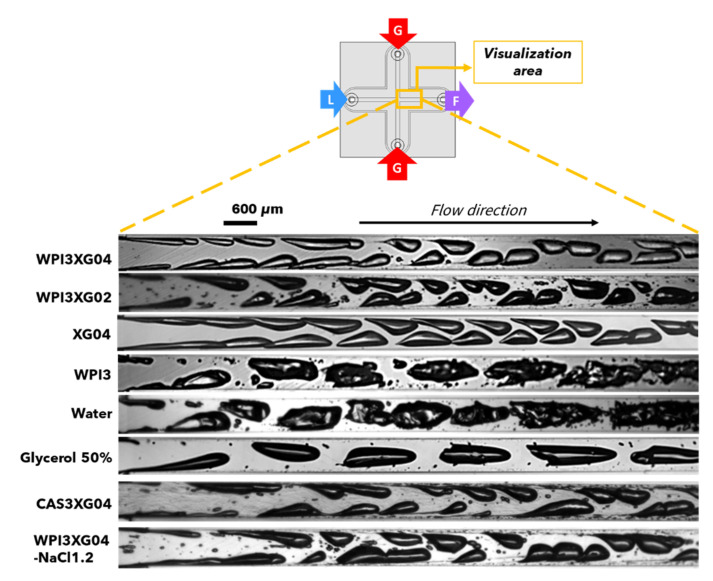
High-speed visualizations of the train of bubbles in the mixing channel of the device CX600 for several formulations at Q_vL_ = 11 L·h^−1^ − Q_mG_ = 21.6 g·h^−1^. The liquid flowrate for CAS3XG04 was slightly lower (Q_vL_ = 10.6 L·h^−1^ − Q_mG_ = 21.6 g·h^−1^).

**Figure 16:**
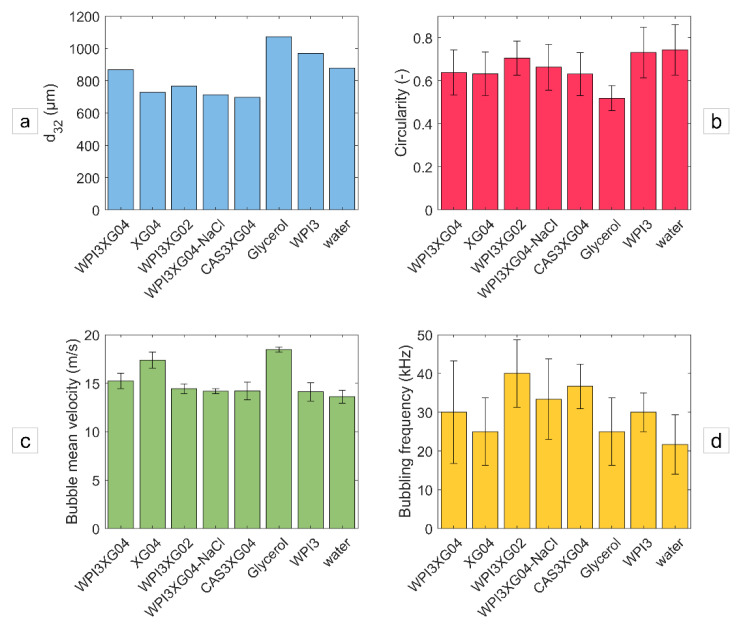
Properties of bubbles flowing through several liquid bases inside the mixing channel of the device CX600 at Q_vL_ = 11 L·h^−1^ − Q_mG_ = 21.6 g·h^−1^. (**a**) Sauter diameter. (**b**) Circularity. (**c**) Average bubble speed. (**d**) Bubbling frequency. The liquid flowrate for CAS3XG04 was slightly lower (conditions for CAS3XG04 were Q_vL_ = 10.6 L·h^−1^ − Q_mG_ = 21.6 g·h^−1^.

This section specifically focuses on the formation of bubble trains and their flow typology in a straight outlet channel (mixing microchannel) using various solutions and fluids. Therefore, experiments are conducted in the device CX600. Figure 15 presents an example of the visualization of the two-phase flow obtained for different formulations in the mixing channel of this device using, as a fixed condition, a liquid flowrate of around 11 L·h^−1^. Several characteristics of the train of bubbles from Figure 15 are estimated (Figure 16). In addition to the previously discussed formulations (i.e., WPI3XG04, WPI3XG02, XG04, WPI3, glycerol and water), two additional solutions were studied in this section, namely WPI3XG04-NaCl1.2 and CAS3XG04. The series of successive images for these two solutions are presented in Figures S3 and S4, along with the already presented series for the solution WPI3XG04 for comparison purposes. WPI3XG04-NaCl1.2 corresponds to one of our model solutions (WPI3XG04), to which 1.2% *w*/*w* (~0.20 M) of sodium chloride is added in order to evaluate whether there is any effect of the ionic strength on the flow phenomena. The selected concentration of NaCl is in the concentration range of 0.05 to 1.0 M, used to promote the solubility of the constituents of a mixture of xanthan gum and whey protein isolates [35]. CAS3XG04 contains 3% *w*/*w* sodium caseinates and 0.4% *w*/*w* xanthan gum. In this case, the purpose is to evaluate the eventual impact of the protein configuration on the interfacial deformation and breakup; WPI are rather globular proteins, while CAS have more open configurations. It is important to point out that due to some technical issues, the liquid flowrate for the solution CAS3XG04 was slightly lower (10.6 L·h^−1^). The estimated characteristics for the bubbles in the mixing channel using these formulations are the Sauter diameter (Figure 16a), circularity (Figure 16b), average bubble velocity (Figure 16c) and bubble frequency (Figure 16d).

Bubbles in solutions with xanthan tend to present smaller values of d_32_ (Figure 16a). Lower inertial forces and the stabilizing role of xanthan previously discussed might explain this result. Concerning circularity (Figure 16b), a decrease in xanthan concentration, represented by WPI3XG02 compared to WPI3XG04, tends to generate slightly more circular bubbles. In general, less viscous solutions (WPI3XG02, WPI3 and water) display higher circularities. However, the surfaces of bubbles flowing through water and WPI3 are extremely irregular. Therefore, a more detailed study of bubble shapes might be beneficial for gaining more insight in this regard. For the average bubble velocity (Figure 16c), xanthan and glycerol show slightly higher average bubble velocities (17 and 18 m·s^−1^, respectively) compared to the other solutions (15 m·s^−1^). The question that arises is whether the excess concentration of proteins at the interface can impact the flow streamlines. This deserves to be further investigated in future research to corroborate its validity. The most significant differences are observed for the bubbling frequency (Figure 16d). In this case, the protein-free solutions exhibit lower bubbling frequency of around 25 kHz for glycerol and XG04, and 20 kHz for water. These values result from the greater interfacial tensions counteracting inertial forces at the moment of bubble detachment during bubble genesis. The liquid Weber numbers, measuring the balance between inertial and interfacial forces, are indeed the lowest for these solutions: 598, 709 and 596 for XG04, glycerol and water, respectively (Table 4, Table 6 and Table 8). A comparison between the solutions containing proteins and xanthan gum seems to indicate a higher bubbling frequency with the lowest concentration of xanthan gum (WPI3XG02). This would result from the higher inertial forces involved, which lead to the rapid detachment of bubbles in this solution; a higher Reynolds number of 3214 was found for WPI3XG02, compared to values in the range of 1800 to 2000 for the solutions WPI3XG04, WPI3XG04-NaCl and CAS3XG04 (Table 4, Table 6 and Table 8).

Finally, Table 9 synthetizes relevant information. Among this information, the values of two ratios are provided for a given gas/liquid flow rate pair, in relation to the case of water. One is concerning the bubble frequency and the other one relates to the Weber number of the liquid. Water is presently used as a reference, because it contains no additives. It can be seen that three formulations stand out in terms of bubbling performance (WPI3XG02, CAS3XG04 and WPI3XG04-NaCl), which are shown in bold. They have in common the presence of a surfactant and a thickening agent, xanthan. This shows the importance of the formulation in the formation step of the bubble train in the mixing channel in high-throughput conditions. As empirically known, an adequate combination of molecules added to a convenient choice of concentrations and flow conditions is necessary to obtain a final foam with convenient properties.

Table 9 also synthetizes the information on relevant breakup phenomena, if observed in this work. These are tip-streaming in the bubbling channel and binary breakup in the expansion section of CX-E-600. The uses of all the three of the most interesting formulations listed above are to allow the development of these phenomena, and they are characterized by a ratio of the number of We_L_ which is at the order of 1.5 to 1.6 (both calculated under the same flow conditions in the bubbling channel).

It would be interesting to study combinations including other thickening agents (guar, alginates…) and natural surfactants in order to determine more thoroughly the effect of the rheological characteristics of thickening polymers and that of the size and shape of surfactants on the bubble train formation in microsystems at high throughputs. However, it may be emphasized that due to pressure drop limitations, it is advisable to develop sophisticated formulations that optimize the respective role of each component. Indeed, this work provides a first glimpse of the effect of the formulation at different steps of a foaming process based on microfluidics at high flow rates.

## 4. Conclusions

This study has investigated the use of microchannels for the production of liquid food foams using model solutions formulated with native whey protein isolates and xanthan gum. The formulation as well as the liquid flowrates and the foam void fraction were selected in accordance with relevant conditions for food industrial applications. Therefore, this study challenged traditional processing conditions used in microfluidics. The results obtained here probably correspond to the first attempt to identify the mutual interactions of the molecules with process parameters. This experimental work is based on visualizations of the breakup mechanisms of gas–liquid interfaces under “extreme” conditions: confined two-phase flow at high throughputs, noticeably short time scales, and complex fluids. Furthermore, from a technical point of view, this research also made it possible to progress in the implementation of an experimental setup and in the selection of the operating parameters and image acquisition conditions suitable for the study of two-phase flow in microchannels at the “extreme” conditions mentioned above.

In this way, this study emphasized the gas–liquid breakup mechanisms underlying the manufacturing of liquid food foams. Two microfluidic devices with different geometries were studied (CX600 and CX-E-600), providing strong experimental evidence on the effect of the device geometry on the gas–liquid interface breakup. The intensification of the bubble breakup identified in the expansion channel of the device CX-E-600 was one of the main results of this research. This observation has to be linked to the potential effect of sudden variations in the cross section of tubes used in relaxation lines.

This study indubitably highlighted the essential role of the interaction process/formulation in the development of the structural and functional properties of food foams when using microfluidics. Studying various formulations allowed us to demonstrate the key role played by the interactions between whey protein isolates or sodium caseinates and xanthan gum while implementing an intensified process that involved high shear rates. Another important contribution of this study was the identification of the mechanisms of bubble formation inside the microchannels according to the rheological and tensioactive properties of the constituents at high shear rates, in the range of 10^3^ to 10^5^ s^−1^. A wide range of properties were indeed investigated in order to contribute to the proposition of scaling laws for the industrial production of foams in these conditions.

## Figures and Tables

**Table 1:** Physico-chemical properties of solutions at 20 °C.

Solution	*σ*	*ρ*
*-*	mN·m^−1^	kg·m^−3^
WPI3XG02	43	1010
WPI3XG04	46	972
XG04	72	1003
WPI3XG04 + NaCl	46	979
CAS3XG04	41	968
WPI3	40	1006
Glycerol 50%	68	1123
Water	72	1000

**Table 2:** Hydrodynamic parameters of the two-phase flow in the mixing channel of the microfluidic device CX600 for several flow conditions using the model solution WPI3XG04.

*P_pump_*	*Q_vL_*	*U_L_*	*Q_mG_*	*p_inj_*	*Q_vG_*	*U_G_*	*α (p)*	$\gamma_{app}^{\cdot }$	*µ_L_*	*Ca_L_*	*Re_L_*	*We_L_*	Δ*P*_1_
bar	L·h^−1^	m·s^−1^	g·h^−1^	bar	L·h^−1^	m·s^−1^	-	s^−1^	mPa·s	-	-	-	bar
2.05	3.0	2.17	5.00	1.53	1.70	1.31	0.38	2.57 × 10^4^	3.38	0.16	373	60	0.48
3.89	6.9	5.32	13.00	2.70	3.02	2.33	0.30	6.30 × 10^4^	2.69	0.31	1153	358	1.00
6.37	11.0	8.46	21.60	3.83	3.85	2.97	0.26	1.00 × 10^5^	2.49	0.46	1981	907	1.53
7.78	13.7	10.56	26.50	4.65	4.04	3.12	0.23	1.25 × 10^5^	2.42	0.56	2545	1413	1.97
9.22	16.4	12.66	31.00	5.52	4.09	3.16	0.20	1.50 × 10^5^	2.37	0.65	3113	2030	2.34
11.81	19.1	14.75	36.40	6.45	4.21	3.25	0.18	1.75 × 10^5^	2.34	0.75	3684	2759	2.90

**Table 3:** Hydrodynamic parameters of the two-phase flow in the expansion channel of the microfluidic device CX-E-600 for several flow conditions using the model solution WPI3XG04.

*Q_vL_*	*U_L_*	*Q_vG_*	*U_G_*	$\gamma_{app}^{\cdot }$	*µ_L_*	*Ca_L_*	*Re_L_*	*We_L_*
L·h^−1^	m·s^−1^	L·h^−1^	m·s^−1^	s^−1^	mPa·s	-	-	-
3.0	0.78	12.87	0.36	5.55 × 10^3^	6.88	0.12	110	13
6.9	1.91	19.83	0.55	1.36 × 10^4^	4.32	0.18	431	77
11.0	3.05	25.28	0.70	2.17 × 10^4^	3.59	0.24	825	196
13.7	3.80	23.21	0.64	2.70 × 10^4^	3.33	0.28	1109	305
16.4	4.56	26.15	0.73	3.24 × 10^4^	3.15	0.31	1405	439

**Table 4:** Hydrodynamic parameters of the two-phase flow in the mixing channel of the microfluidic device CX600 for the XG04, WPI3XG04 and glycerol solutions at Q_vL_ = 11 L·h^−1^ − Q_mG_ = 21.6 g·h^−1^.

*Solution*	*Q_vL_*	*U_L_*	*p_inj_*	*Q_vG_*	*U_G_*	$\gamma_{app}^{\cdot }$	*µ_L_*	*Ca_L_*	*Re_L_*	*We_L_*	Δ*p*_1_
-	L·h^−1^	m·s^−1^	bar	L·h^−1^	m·s^−1^	s^−1^	mPa·s	-	-	-	bar
XG04	11.0	8.46	3.53	4.16	3.21	1.00 × 10^5^	2.14	0.25	2379	598	1.58
WPI3XG04	11.0	8.46	3.83	3.85	2.97	1.00 × 10^5^	2.49	0.46	1981	907	1.53
Glycerol	11.0	8.46	4.60	3.37	2.60	1.00 × 10^5^	6.00	0.75	950	709	2.25

**Table 5:** Hydrodynamic parameters of the two-phase flow in the expansion channel of the microfluidic device CX-E-600 for the XG04, WPI3XG04 and glycerol solutions at Q_vL_ = 3 L·h^−1^ − Q_mG_ = 5 g·h^−1^.

*Solution*	*Q_vL_*	*U_L_*	*Q_vG_*	*U_G_*	$\gamma_{app}^{\cdot }$	*µ_L_*	*Ca_L_*	*Re_L_*	*We_L_*
(-)	L·h^−1^	m·s^−1^	L·h^−1^	m·s^−1^	s^−1^	mPa·s	-	-	-
XG04	3.0	0.80	1.51	0.42	5.66 × 10^3^	4.94	0.05	162	9
WPI3XG04	3.0	0.78	12.87	0.36	5.55 × 10^3^	6.88	0.12	110	13
Glycerol	3.0	0.83	1.26	0.35	5.87 × 10^3^	6.00	0.07	154	11

**Table 6:** Hydrodynamic parameters of the two-phase flow in the mixing channel of the microfluidic device CX600 for the solutions WPI3, WPI3XG02 and water at Q_vL_ = 11 L·h^−1^ − Q_mG_ = 21.6 g·h^−1^.

*Solution*	*Q_vL_*	*U_L_*	*p_inj_*	*Q_vG_*	*U_G_*	$\gamma_{app}^{\cdot }$	*µ_L_*	*Ca_L_*	*Re_L_*	*We_L_*	Δ*p_1_*
-	L·h^−1^	m·s^−1^	Bar	L·h^−1^	m·s^−1^	s^−1^	mPa·s	-	-	-	bar
WPI3	11.0	8.46	3.34	4.31	3.33	1.00 × 10^5^	1.44	0.30	3548	1079	1.46
WPI3XG02	11.0	8.46	3.08	4.61	3.56	1.00 × 10^5^	1.52	0.29	3214	945	13.46
Water	11.0	8.46	2.49	5.35	4.13	1.00 × 10^5^	1.00	0.12	5076	596	1.46

**Table 7:** Hydrodynamic parameters of the two-phase flow in the expansion channel of the microfluidic device CX-E-600 for the solutions WPI3, WPI3XG02 and water at Q_vL_ = 3 L·h^−1^ − Q_mG_ = 5 g·h^−1^.

*Solution*	*Q_vL_*	*U_L_*	*Q_vG_*	*U_G_*	$\gamma_{app}^{\cdot }$	*µ_L_*	*Ca_L_*	*Re_L_*	*We_L_*
(-)	L·h^−1^	m·s^−1^	L·h^−1^	m·s^−1^	s^−1^	mPa·s	-	-	-
WPI3	3.0	0.77	1.80	0.50	5.45 × 10^3^	1.44	0.03	536	15
WPI3XG02	3.0	0.78	1.54	0.43	5.55 × 10^3^	3.73	0.07	201	13
Water	3.0	0.83	2.04	0.57	5.93 × 10^3^	1.00	0.01	833	10

**Table 8:** Hydrodynamic parameters of the two-phase flow in the mixing channel of the microfluidic device CX600 for the solutions WPI3XG04-NaCl at Q_vL_ = 11 L·h^−1^ − Q_mG_ = 21.6 g·h^−1^ and CAS3XG04 at Q_vL_ = 10.6 L·h^−1^ − Q_mG_ = 21.6 g·h^−1^.

*Solution*	*Q_vL_*	*U_L_*	*p_inj_*	*Q_vG_*	*U_G_*	$\gamma_{app}^{\cdot }$	*µ_L_*	*Ca_L_*	*Re_L_*	*We_L_*	Δ*p*_1_
-	L·h^−1^	m·s^−1^	bar	L·h^−1^	ms^−1^	s^−1^	mPa·s	-	-	-	bar
CAS3XG04	10.6	8.15	4.54	3.38	2.61	9.67 × 10^4^	2.55	0.51	1856	939	1.89
WPI3XG04-NaCl	11.0	8.46	3.84	3.85	2.97	1.36 × 10^5^	2.43	0.45	2048	914	1.51

**Table 9:** Qualitative summary of the effect of the formulation on the two-phase flow in the mixing channel of the device CX600. The ratios concern the flow in the mixing channel; they are calculated at Q_vL_ = 11 L·h^−1^ − Q_mG_ = 21.6 g·h^−1^. The liquid flowrate for CAS3XG04 was slightly lower (Q_vL_ = 10.6 L·h^−1^ − Q_mG_ = 21.6 g·h^−1^). The indications “yes” and “no” in the 4th and 5th columns refer to the visual presence or absence of bubble breakup mechanisms discussed in this study: i.e., tip-streaming in the mixing channel and binary breakup in the expansion channel of CX-E-600. The last column indicates if the solution/fluid allowed us to obtain a foam or not.

Solution/Fluid	f/f_water_	We_L_/We_Lwater_	Tip Streaming	Binary Breakup	Foam at the Outlet
WPI3XG04	1.43	1.52	yes	yes	yes
**WPI3XG02**	**1.90**	**1.58**	**yes**	**yes**	**yes**
XG04	1.14	1.00	no	no	no
WPI3	1.43	1.81	no	no	Yes but rough and unstable
Glycerol	1.19	1.19	no	no	no
Water	1.00	1.00	no	no	no
**CAS3XG04**	**1.71**	**1.57**	**probable**	**yes**	**yes**
**WPI3XG04-NaCl**	**1.62**	**1.53**	**yes**	**yes**	**yes**

## Supplementary Materials

The following are available online at https://www.mdpi.com/article/10.3390/mi12111415/s1. Figure S1: Series of successive high-speed images of the two-phase flow inside the mixing channel of the device CX600. Flowrates used: *Q**_vL_* = 11 L·h^−1^ et *Q**_mG_* = 21.6 g·h^−1^. (**a**) solution WPI3XG02. (**b**) solution WPI3. (**c**) tap water. Image acquisition frequency of 5000 images per second resulting in interframe time of 0.2 ms; Figure S2: Series of high-speed images selected at an interval of 0.8 ms for the two-phase flow inside the expansion channel of the device CX-E-600. Flowrates used: *Q**_vL_* = 3 L·h^−1^ et *Q**_mG_* = 5 g·h^−1^. (**a**) solution WPI3XG02. (**b**) solution WPI3. (**c**) tap water. Image acquisition frequency of 5000 images per second resulting in interframe time of 0.2 ms; Figure S3: Series of successive high-speed images of the two-phase flow inside the mixing channel of the device CX600. Flowrates used: *Q**_vL_* = 11 L·h^−1^ et *Q**_mG_* = 21.6 g·h^−1^ for WPI3XG04 and WPI3XG04-NaCl and *Q**_vL_* = 10.6 L·h^−1^ et *Q**_mG_* = 21.6 g·h^−1^ for CAS3XG04. (**a**) solution WPI3XG04. (**b**) solution CAS3XG04. (**c**) solution WPI3XG04-NaCl. Image acquisition frequency of 5000 images per second resulting in interframe time of 0.2 ms; Figure S4: Series of high-speed images selected at an interval of 0.8 ms for the two-phase flow inside the expansion channel of the device CX-E-600. Flowrates used: *Q**_vL_* = 3 L·h^−1^ et *Q**_mG_* = 5 g·h^−1^ for WPI3XG04 and WPI3XG04-NaCl and *Q**_vL_* = 2.6 L·h^−1^ et *Q**_mG_* = 5 g·h^−1^ for CAS3XG04. (**a**) solution WPI3XG04. (**b**) solution CAS3XG04. (**c**) solution WPI3NaCl. Image acquisition frequency of 5000 images per second resulting in interframe time of 0.2 ms.

## Nomenclature

Latin symbols	
a	Curvature parameter
B_b_	Largest radial dimension of a bubble
d	Diameter of a bubble
$d_{\mathrm{eq}}$	Equivalent diameter of a sphere
$d_{32}$	Sauter diameter
$D_{h}$	Hydraulic diameter
G’	Elastic modulus
G’’	Viscous modulus
H	Foam height
H_0_	Initial foam height
K_s_	Geometric constant of channels
L_b_	Largest axial dimension of a bubble
m	Mass
n	Structure index in the Carreau-Yasuda law
p	Pressure
p_inj_	Injection pressure
Q	Flowrate
U	Superficial velocity
V	Bubble volume
$V_{\mathrm{obj}}$	Volume of a solid cylinder used for density determinations
W	Channel width
x	Mass measurement
Devices	
CX600	Cross-shaped channels with hydraulic diameter of 600 µm
CX-E-600	Cross-shaped channels with hydraulic diameter of 600 µm and an integrated expansion of 1000 µm hydraulic diameter
Greek symbols	
${\dot{\gamma }}_{app}$	Process shear rate
λ	Rheological relaxation time
μ	Dynamic viscosity
$\mu_{o}$	1st Newtonian plateau viscosity
$\mu_{\infty }$	2nd Newtonian plateau viscosity
α	Void fraction
ρ	Density
σ	Surface tension
ΔP	Pressure loss
Subscripts	
atm	Ambient
G	Gas phase
L	Liquid phase
m	Mass
v	Volume
R	Reference
Dimensionless numbers	
Ca	Capillary number
Re	Reynolds number
We	Weber number
