# Supplementary material for: Towards High Throughput Structuring of Liquid Foams in Microchannels: Effect of Geometry, Flowrate and Formulation
Source: Micromachines (Basel). 2021 Nov 18;12(11):1415. doi: 10.3390/mi12111415 (PMC8617920; doi:10.3390/mi12111415)
Supplement: Supplementary file 1 [file micromachines-12-01415-s001.zip › micromachines-1434175-supplementary.pdf]

Article

Towards High Throughput Structuring of Liquid Foams in Microchannels: Effect of Geometry, Flowrate and Formulation

Julian Sepulveda [1], Agnès Montillet [1,*], Dominique Della Valle [2], Catherine Loisel [2] and Alain Riaublanc [3]

Supplementary Materials

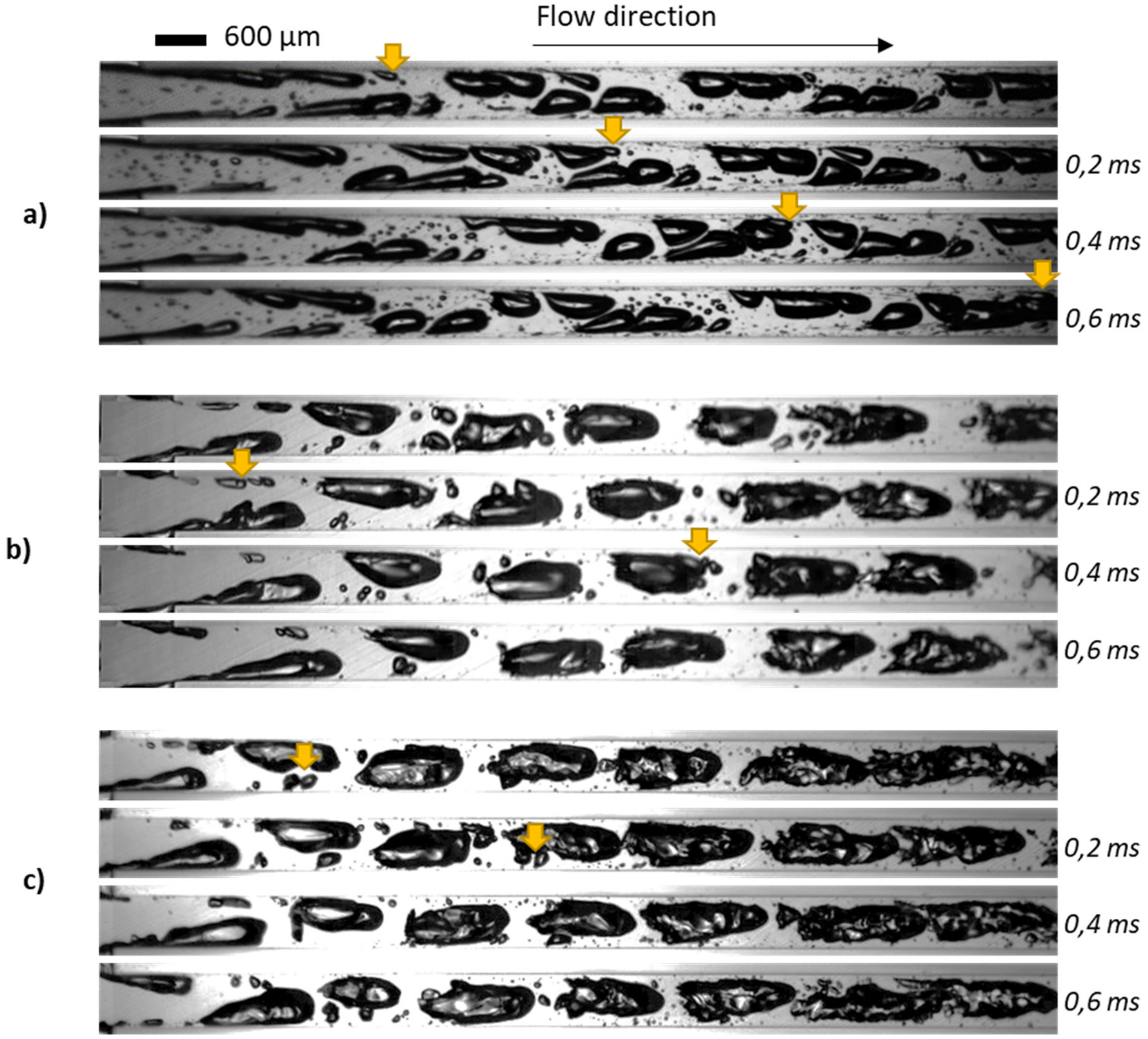

Figure S1. Series of successive high-speed images of the two-phase flow inside the mixing channel of the device CX600. Flowrates used: Q_{vL} = 11 L.h^{-1} et Q_{mG} = 21.6 g.h^{-1}. (**a**) solution WPI3XG02. (**b**) solution WPI3. (**c**) tap water. Image acquisition frequency of 5000 images per second resulting in interframe time of 0.2 ms.

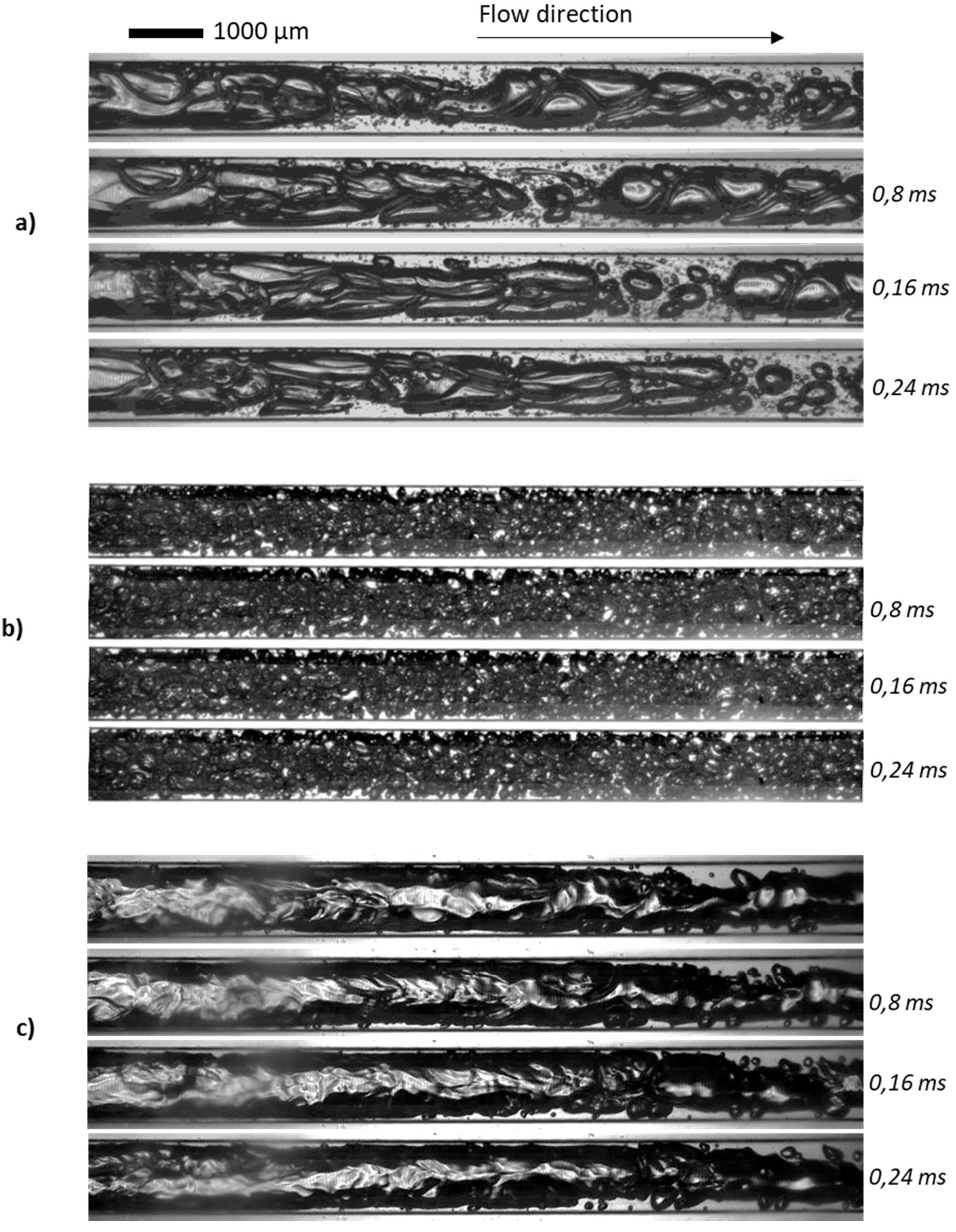

Figure S2. Series of high-speed images selected at an interval of 0.8 ms for the two-phase flow inside the expansion channel of the device CX-E-600. Flowrates used: Q_{vL} = 3 L.h^{-1} et Q_{mG} = 5 g.h^{-1}. (**a**) solution WPI3XG02. (**b**) solution WPI3. (**c**) tap water. Image acquisition frequency of 5000 images per second resulting in interframe time of 0.2 ms.

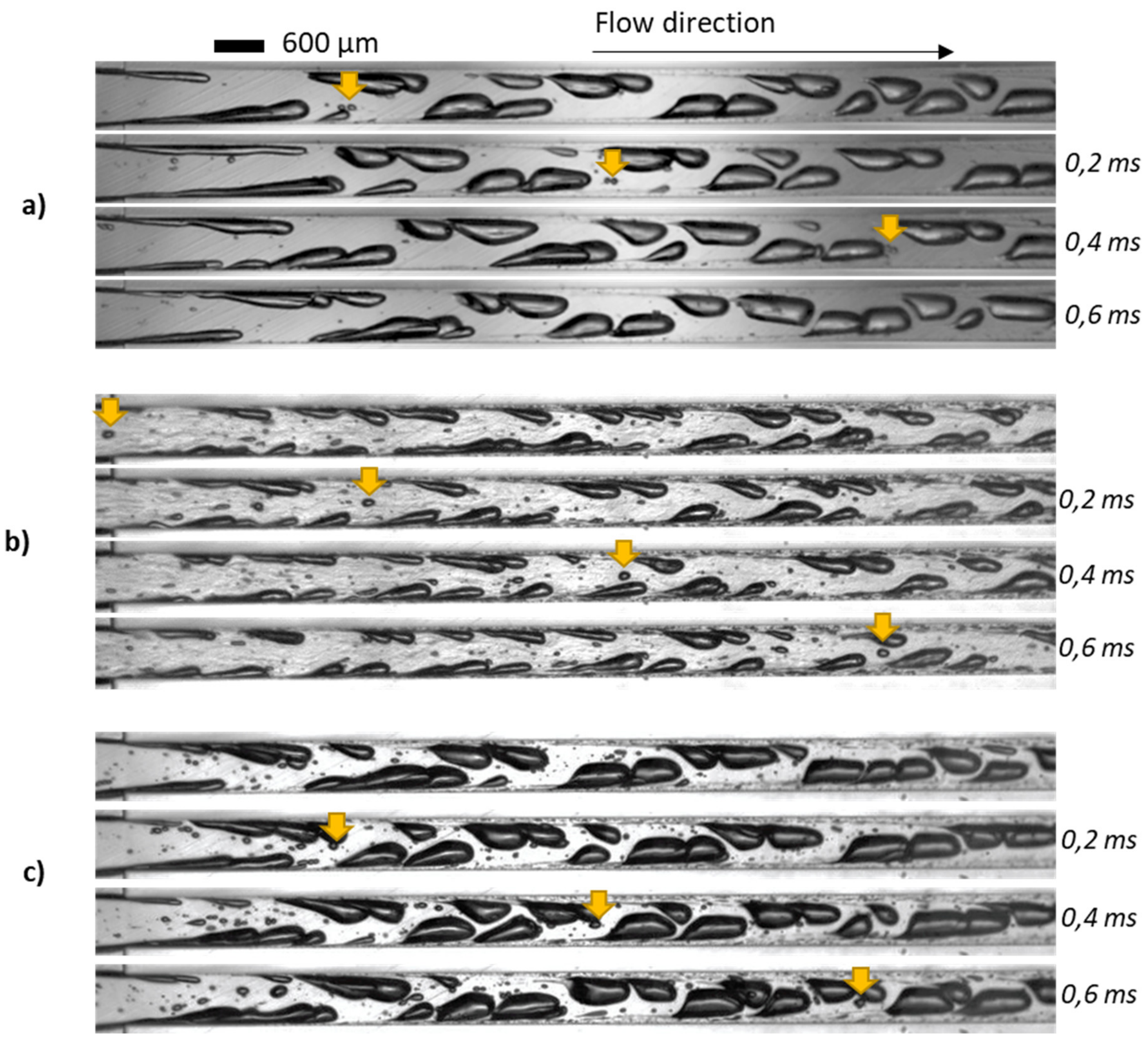

Figure S3. Series of successive high-speed images of the two-phase flow inside the mixing channel of the device CX600. Flowrates used: Q_{vL} = 11 L.h^{-1} et Q_{mG} = 21.6 g.h^{-1} for WPI3XG04 and WPI3XG04-NaCl and Q_{vL} = 10.6 L.h^{-1} et Q_{mG} = 21.6 g.h^{-1} for CAS3XG04. (**a**) solution WPI3XG04. (**b**) solution CAS3XG04. (**c**) solution WPI3XG04-NaCl. Image acquisition frequency of 5000 images per second resulting in interframe time of 0.2 ms.

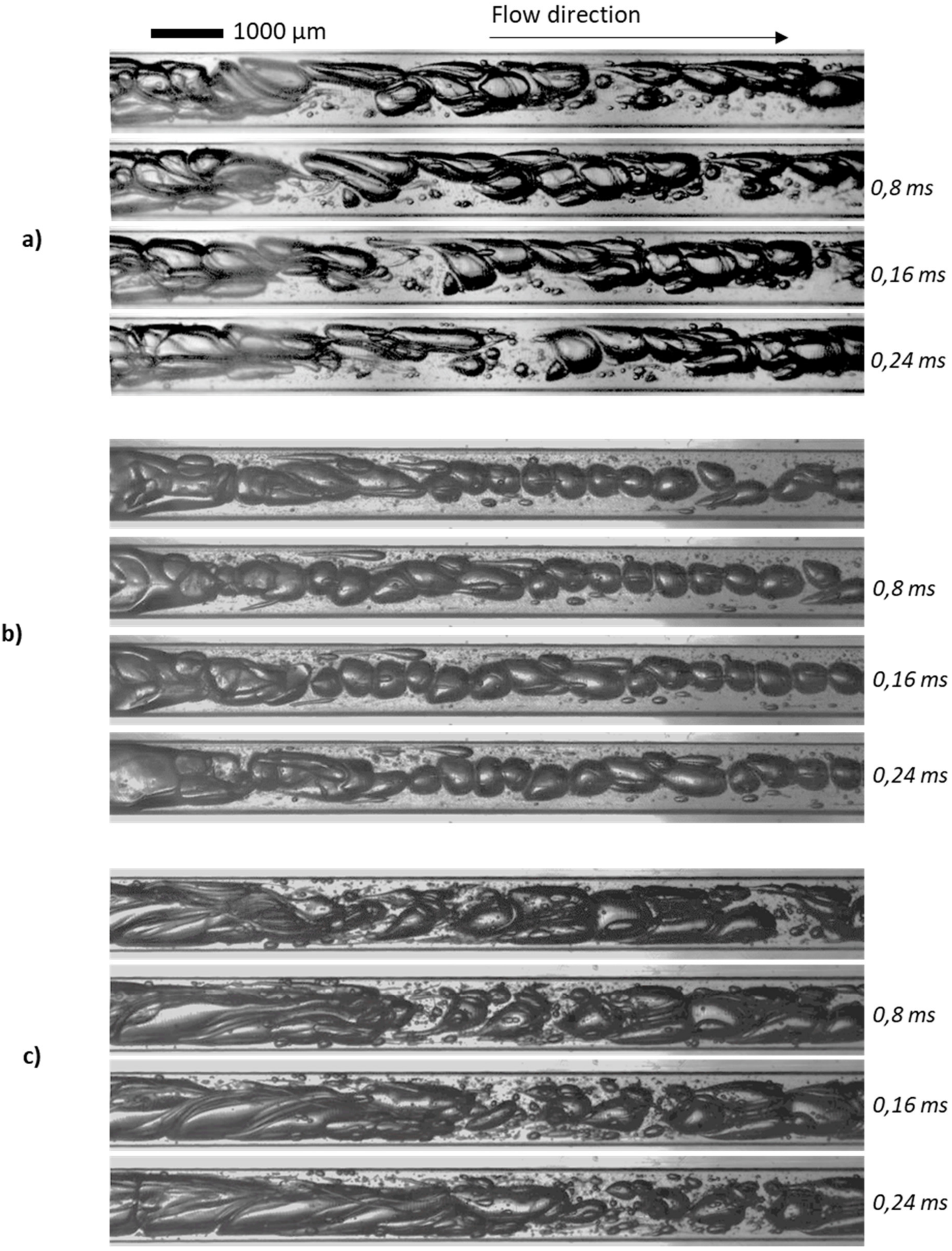

Figure S4. Series of high-speed images selected at an interval of 0.8 ms for the two-phase flow inside the expansion channel of the device CX-E-600. Flowrates used: Q_{vL} = 3 L.h^{-1} et Q_{mG} = 5 g.h^{-1} for WPI3XG04 and WPI3XG04-NaCl and Q_{vL} = 2.6 L.h^{-1} et Q_{mG} = 5 g.h^{-1} for CAS3XG04. (**a**) solution WPI3XG04. (**b**) solution CAS3XG04. (**c**) solution WPI3NaCl. Image acquisition frequency of 5000 images per second resulting in interframe time of 0.2 ms.
